# Isolation and identification of BRV G6P[1] strain in Heilongjiang province, Northeast China

**DOI:** 10.3389/fvets.2024.1416465

**Published:** 2024-09-20

**Authors:** Chunqiu Li, Xiaoran Wang, Qinghe Zhu, Dongbo Sun

**Affiliations:** Heilongjiang Provincial Key Laboratory of the Prevention and Control of Bovine Diseases, College of Animal Science and Veterinary Medicine, Heilongjiang Bayi Agricultural University, Daqing, China

**Keywords:** bovine rotavirus, isolation, identification, phylogenetic analysis, pathogenicity

## Abstract

Bovine rotavirus (BRV) is the main cause of acute gastroenteritis in calves, resulting in significant economic losses to the cattle industry worldwide. Additionally, BRV has multiple genotypes, which could enable cross-species transmission, thereby posing a significant risk to public health. However, there is a problem of multiple genotypes coexisting in BRV, and the cross-protection effect between different genotypes of rotavirus strains is not effective enough. Therefore, mastering clinical epidemic genotypes and using epidemic genotype strains for vaccine preparation is an effective means of preventing and controlling BRV. In this study, BRV strain DQ2020 in MA104 cells was identified by transmission electron microscopy (TEM), reverse transcription polymerase chain reaction (RT-PCR), and colloidal gold immunochromatographic test strips. The whole genome of BRV strain DQ2020 was sequenced and pathogenicity in suckling mice was assessed. The results showed that after 10 passages in MA104 cells, BRV strain DQ2020 induced cytopathic effects. Wheel-shaped virus particles (diameter, ~80 nm) were observed by TEM. A target band of 382 bp was detected by RT-PCR, a positive band was detected with the colloidal gold immunochromatographic test strips, and significant green fluorescence was observed by indirect immunofluorescence (IFA). The highest median tissue culture infectious dose of strain DQ2020 after 9 passages in MA104 cells was 10^−4.81^ viral particles/0.1 mL. Based on phylogenetic analysis of 11 gene fragments, the genotype of BRV strain DQ2020 was G6-P[1]-I2-R2-C2-M2-A11-N2-T6-E2-H3, confirming transmission of the G6-P[1] genotype in Chinese cattle herds. Further analysis showed that the isolated strain was a reassortant of bovine (VP7, VP6, NSP3, and NSP5), human (VP4, VP1, VP2, VP3, NSP2, and NSP4), and ovine (NSP1) rotaviruses. BRV strain DQ2020 caused damage to the intestinal villi of suckling mice and diarrhea, confirming pathogenicity. In summary, this study identified a reassortant strain of bovine, human, and ovine rotavirus that is pathogenic to lactating mice, and conducted whole genome sequence analysis, providing valuable insights for the genetic evolution of the virus and the development of vaccines.

## Introduction

Rotaviruses cause diarrhea in young children and animals worldwide with particularly high incidences in developing countries in Asia and Africa (1). Bovine rotavirus (BRV), either alone or in combination with other viruses or pathogens, is the main cause of diarrhea in bovine species. BRV infection of calves is characterized by diarrhea, depression, anorexia, dehydration, and even acidosis. The incidence of diarrhea in calves linked to BRV infection varies among countries from 7 to 94% with a rate of 46% in China (2–8). The infection of BRV mostly occurs in calves less than 1 month old. Among them, calves less than 7 days old are most likely to be infected. They usually have severe diarrhea within 12 to 24 h after infection. The incidence rate is 90 to 100%, and the highest case fatality rate is 50% (9).

BRV belongs to the family Reoviridae and the genus Rotavirus. The BRV genome consists of 11 segments of double stranded RNA, which encode six structural and five non-structural proteins. The 11 gene fragments of BRV are prone to point mutations, leading to the emergence of new strains and even potential interspecies transmission, thereby expanding the range of hosts (10–14). Based on antigen typing of the outer shell proteins VP4 and VP7 (GxP[x]), epidemic strains of BRV are classified into five G genotypes (G1, G6, G8, G10, and G15) and three P genotypes (P1, P5 and P11). The prevalent strains of BRV in China include the combination genotypes G6P[1], G6P[5], and G10P[11] (12, 15, 16).

Research has shown that cross protection between different genotypes of rotavirus strains is not effective, with a protection rate of only 23–85% (17). Therefore, mastering clinical epidemic genotypes and using epidemic genotype strains for vaccine preparation is an effective means of preventing and controlling BRV. The aim of the present study was to sequence the genotypes of epidemic strains of BRVs isolated from fecal samples of calves with clinical diarrhea to develop new vaccines. In addition, the pathogenicity of the isolated strains was assessed to elucidate the relationships among different BRV strains and potential of transmission to other species.

## Methods

### Ethics statement

This animal experiments complied with the Laboratory Animals Guideline of Welfare and Ethics published by the General Administration of Quality Supervision, Inspection, and Quarantine of the People’s Republic of China. The proposals for the animal experiments were approved by the Ethical Committee for Animal Experiments of Heilongjiang Bayi Agricultural University (approval numbers: DWKJXY2022008).

### Sample collection and processing

In total, 21 fecal samples or anal swabs were collected from calves aged <24 weeks following an outbreak of diarrhea at a cattle farm located in Qiqihar City (Heilongjiang Province, China) in 2020. The fecal samples and anal swabs were shipped on dry ice to our laboratory and stored at −80°C until assayed. The fecal samples were diluted (1:10) with high glucose Dulbecco’s Modified Eagle’s Medium (DMEM) (Gibco, Waltham, MA, United States) and centrifuged at 300 × g for 10 min. The supernatant was collected further centrifuged at 8,000 × g for 30 min and identified by RT-PCR.

### Viral isolation and identification

BRV-positive fecal samples collected in China in 2020 were chosen for viral isolation. BRV was isolated from the fecal sample in accordance with the methods described by Zhu et al. (17) and Elkady et al. (9). The supernatants were filtered through a polytetrafluoroethylene syringe filter (pore diameter, 0.45 μm). Bovine trypsin (Sigma-Aldrich Corporation, St. Louis, MO, United States) was added to the sample at 10 μg/mL for 1 h. African green monkey epithelial (MA 104) cells were washed three times with prewarmed serum-free DMEM and then inoculated with the trypsin-treated samples. After incubation for 60–90 min at 37°C under a humidified atmosphere of 5% CO_2_/95% air, the inoculum was removed and the cells were washed with pre-warmed serum-free DMEM and incubated at 37°C under a humidified atmosphere of 5% CO_2_/95% air. Upon observation of a cytopathic effect, the viruses were isolated from the culture supernatant for serial passages. The isolated viruses were identified by transmission electron microscopy (TEM) and with the use of an indirect immunofluorescence assay (IFA). For the IFA, MA104 cells were inoculated with BRV isolates at a multiplicity of infection of 0.1 for 36 h and then fixed with paraformaldehyde (4%). After blocking with 5% skim milk for 2 h at room temperature, the cells were incubated with primary monoclonal antibodies (dilution, 1:500) against BRV VP6 (Abnova Corporation, Taipei City, Taiwan) for 1 h at 37°C, followed by a fluorescein isothiocyanate-conjugated goat anti-mouse antibody against immunoglobulin G (dilution, 1:2,000; Anolun (Beijing) Biotechnology Co., Ltd., Beijing, China). The cells were then observed under a microscope. For TEM, the samples were negatively stained as previously described by Jiang et al. (18). The viral suspension was centrifuged at 30,000 × g for 30 min. The resulting pellet of viral particles was negatively stained with 2% phosphotungstic acid (pH 7.0) and examined using a transmission electron microscope (model 7,650; Hitachi High-Technologies Corporation, Tokyo, Japan). The isolated viruses were identified by RT–PCR and qPCR. RT–PCR with the One-step RT–PCR Kit (TaKaRa, Dalian, China) and specific primers (BRV-VP6-F: 5′-TTGATGGGTACGATGTGGCT-3′; BRV-VP6-R: 5′-CTGGTGTCATATTTGGTGGTCT-3′) to amplify the partial sequence (382 bp of the BRV genome). The BRV isolate was named strain DQ2020.

### Virus purification and growth curve

BRV strain DQ2020 was purified by plaque cloning in MA104 cells. Briefly, Bovine trypsin was added to BRV strain DQ2020 at 10 μg/mL for 1 h. MA104 cells were incubated with serially diluted BRV strain DQ2020 in post-inoculation medium at 37°C for 90 min under atmosphere of 5% CO_2_. Afterward, the medium was removed and the cells in each well were overlaid with 2 mL of post-inoculation medium containing 1.5% agarose. After the overlaid medium had solidified, the cells were grown at 37°C under an atmosphere of 5% CO_2_ to promote plaque formation. Macroscopically visible plaques were picked with a pipette pick, dissolved in 1 mL of DMEM, frozen and thawed for three times after receiving poison, and then inoculated in MA104 cells for propagation in preparation for the next round of plaque purification. The plaque purification operation was repeated five times. After incubation for 48 h, the agarose was covered. When plaques grew to the proper size, the cells were fixed in paraformaldehyde (4%) for 2 h and the plaques were visualized.

Viral growth curves of BRV strain DQ2020 in MA104 cells was constructed according to the median tissue culture infective dose (TCID_50_). Briefly, MA104 cells were seeded in the wells of 96-well plates at a density of 10^5^ cells per well in 100 μL of medium and incubated for 48 h at 37°C under an atmosphere of 5% CO_2_. Afterward, the medium was removed and 100 μL of 10-fold serial dilutions of the virus were added to each well. The cytopathic effect was examined every 12 h for 7 days post-inoculation. The viral titer was determined according to the Reed and Muench method (19).

### Next-generation sequencing

In order to obtain the entire genome of the identified BRV DQ2020 strains, the cDNAs of cell culture medium supernatant sample was sequenced by Illumina next-generation sequencing (Shanghai Tanpu Biotechnology Co., Ltd., Shanghai, China). In brief, viral RNA was isolated and reverse-transcribed into complementary DNA (cDNA) in accordance with the protocol described by Wang et al. (20) using random primers (six nucleotides) and an oligo dT18 primer. Double-stranded DNA was synthesized with a Second Strand cDNA Synthesis Kit (Beyotime Institute of Biotechnology, Shanghai, China). Subsequently, a library was constructed from 150 ng of cDNA using the NEBNext^®^ Ultra™ II RNA Library Prep Kit (New England Biolabs, Ipswich, MA, United States). After adapter ligation, the sequencing target was amplified by 10 cycles of real-time quantitative polymerase chain reaction (RT-qPCR). The libraries were pooled in equimolar amounts, denatured, and diluted to the optimal concentration before sequencing. The Illumina NovaSeq 6,000 System was used for sequencing to generate 150-bp pair-end reads. The trimmed reads were assembled using SOAPdenovo v2.04^1^ and the assembled genomes were corrected using GapCloser v1.12 software.^2^ Viral genes were predicted using GeneMarkS software^3^ and annotated using the Basic Local Alignment Search Tool (BLAST)^4^ in reference to the UniProt non-redundant protein database.^5^ In this study, the complete genome of BRV strain DQ2020 was obtained. After genome assembly, the single genomic gap of BRV strain DQ2020 was closed using standard Sanger sequencing technology. A similarity plot of the genome of BRV strain DQ2020 was constructed with SimPlot v.3.5.1 software (21) using the sliding window method.

### Sequence analysis

The open reading frames, amino acid translation, sequence alignment, and pairwise sequence comparisons were performed using the EditSeq and MegAlign modules of the Lasergene 12 Core Suite (DNASTAR, Inc., Madison, WI, United States). The greatest similarities between the studied BRV genome segments (VP1, VP2, VP3, VP4, VP6, VP7, NSP1, NSP2, NSP3, NSP4, and NSP5) and the published sequences were identified using the BLASTn algorithm.^6^ Multiple sequence alignments were performed using the multiple sequence alignment algorithm of DNAMAN 6.0 software (Lynnon BioSoft, Point-Claire, Quebec, Canada).

### Phylogeny analysis

For genotyping the BRV strain BRV DQ2020, we used the Standard Local Alignment Search tool on the NCBI website to extract sequences identical to each of the 11 protein segments (VP1, VP2, VP3, VP4, VP6, VP7, NSP1, NSP2, NSP3, NSP4 and NSP5) of our strains under analysis. Sequences identical to each of the 11 protein segments of BRV strain DQ2020 (VP1, VP2, VP3, VP4, VP6, VP7, NSP1, NSP2, NSP3, NSP4, and NSP5) were retrieved from the National Center for Biotechnology Information (NCBI) nucleotide database (Supplementary Table S1) and aligned with the ClustalX v.1.83 multiple sequence alignment algorithm.^7^ Phylogenetic trees of all target sequences were constructed using Molecular Evolutionary Genetics Analysis 6.06 software with the neighbor-joining method and 1,000 bootstrap replicates (22). The phylogenetic trees were pruned and re-rooted using Interactive Tree Of Life software version 4.2.3,^8^ which is an online tool for displaying circular trees and annotations (23). Additionally, similarity plots of the genomes of the BRV strains identified in the present study were created using SimPlot v.3.5.1 software (21) using the sliding window method.

### Pathogenicity of BRV DQ2020 in suckling mice

#### Animal experiment design

As described by Rey et al. (24), specific pathogen-free Kunming mice (*n* = 18; age, 7 days) were allocated to one of two group (*n* = 9 each) in separate cages: BRV infection group (oral gavage with 0.1 mL of Dulbecco’s modified Eagle’s medium containing 10^−4.81^ TCID_50_ of BRV strain DQ2020) or a control group (oral gavage with 0.1 mL of DMEM). Through oral gavage to challenge the poison. The mice were observed daily. Three mice from each group were sacrificed every 3 days. Tissues were collected for histopathological analysis and BRV detection by RT-qPCR.

#### Clinical assessment

The mice body temperatures and weights were recorded before inoculation (−12 hpi) and then every 12 h until death. All mice were evaluated twice daily for clinical signs of diarrhea (25). Diarrhea severity was scored according to the following criteria: 0 = normal; 1 = soft (“cow pies”); 2 = very soft and tended to be liquid; 3 = liquid with some solid content; 4 = watery diarrhea with no solid content. In addition, the mortality of the suckling mice in each group was recorded twice daily.

#### Measurement of viral load in tissues

The heart, liver, spleen, lung, kidney, small intestine, rectum, and stomach tissues (0.1 g) were ground in sterile DMEM at a ratio of 1:3 and centrifuged 8,000 × g for 10 min at 4°C. The supernatant was collected for RNA extraction and reverse transcription into cDNA, as described by Wang et al. (12). The BRV VP6 gene sequence was amplified by relative RT-qPCR with the primer pair 5´-ATTGTCGAAGGCACATTATAC-3′/5´-ATTGTATTGCGGGCCGTTTCG-3′ with SYBR Green I fluorescent dye and a QuantStudio™ 3 Real-Time PCR System (Applied Biosystems, Carlsbad, CA, United States), as described by Wang et al. (26). The mice β-actin sequence was amplified with the primer pair 5´-GTCAGGTCATCACTATCGGCAAT-3′/5´-AGAGGTCTTTACGGATGTCAACGT-3′. Each 20 μL reaction included 10 μL of 2 × SYBR^®^ Premix Ex Taq polymerase (Takara Bio, Inc., Shiga, Japan), 2 μL of cDNA, and 0.25 μM of each primer. The thermal cycling conditions included a denaturation step at 95°C for 30 s, followed by 40 cycles at 95°C for 25 s and 60°C for 60 s. Ten-fold dilutions of a standard plasmid (10^−10^–10^1^) and the negative control (distilled water) were included in each plate. Each sample was assayed three times. The viral RNA was quantified in reference to the standard plasmid.

#### Histopathology analysis

Three days after infection with BRV strain DQ2020, the mice were dissected and small intestine tissues were collected for histopathological analysis. The tissue samples were fixed in 10% formalin at room temperature for 48 h and embedded in paraffin. Prior to histopathological analysis, the tissues were dewaxed with xylene and anhydrous ethanol, mounted on glass slides, washed, stained with hematoxylin and eosin, sealed with neutral gum, and observed under a microscope at 100× magnification.

### Statistical analysis

The data are presented as the mean ± standard deviation of experiments conducted in triplicate. All statistical analyses (one-way analysis of variance and the Student’s *t*-test) were conducted using Prism 8.0 software (GraphPad Software, Inc., La Jolla, CA, United States). Probability (p) values of <0.05 and < 0.01 were considered statistically significant and highly significant, respectively. **** indicates that the difference was extremely significant.

## Results

### Viral isolation and identification

**Figure 1:**
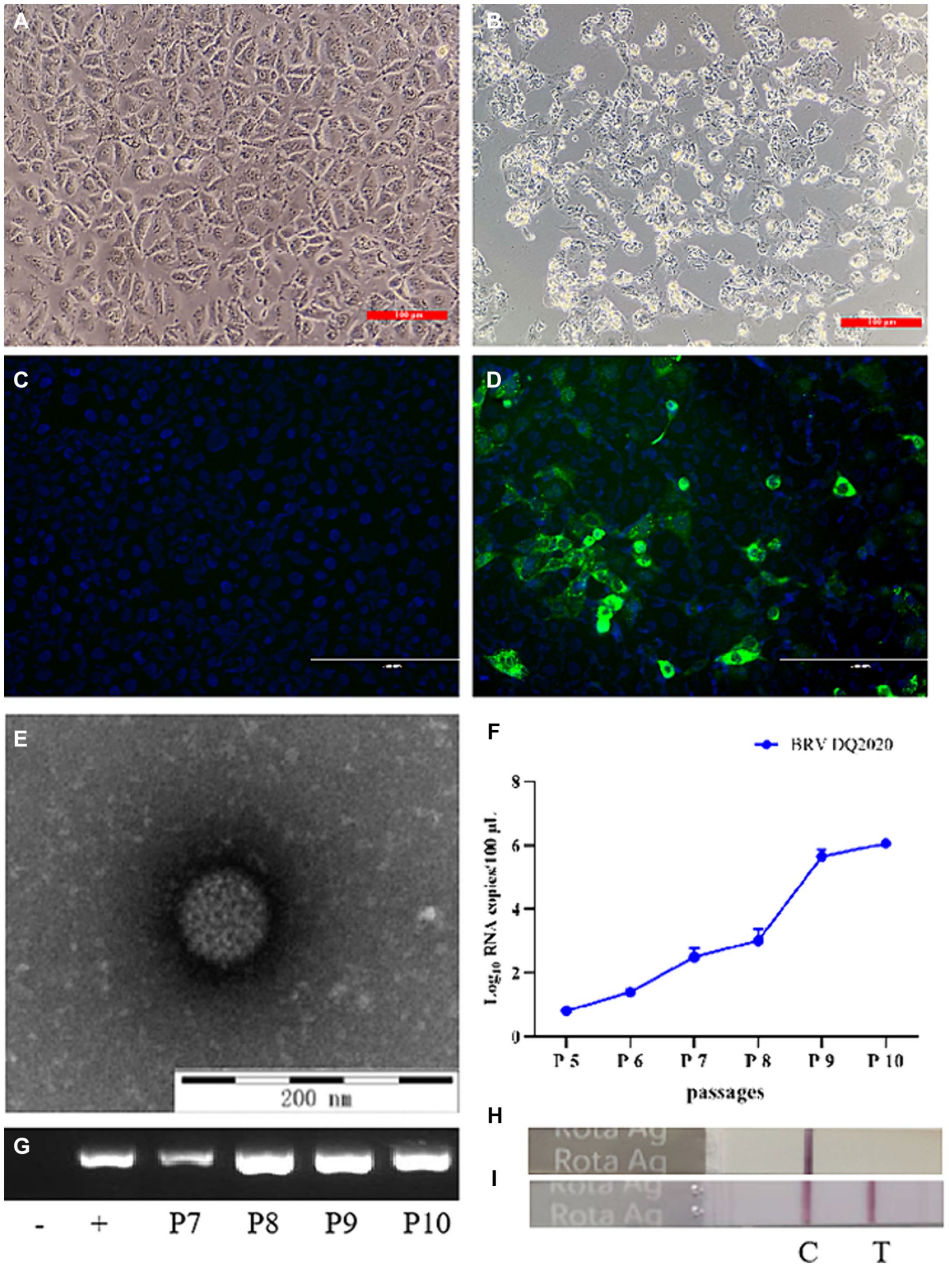
Identification of the isolated BRV DQ2020 strain. **(A)** Mock-inoculated MA104 cells (100×). **(B)** At 48 h post-inoculation, MA104 cells showed cytopathic changes, such as rounding, aggregation, and shedding (100×). **(C)** Non-infected MA104 cells did not emit fluorescence. **(D)** MA104 cells inoculated with BRV strain DQ2020 for 36 h emitted green fluorescence (200×). **(E)** Typical rotavirus particles (diameter, 80 nm) were observed in the supernatant of MA104 cells inoculated with BRV strain DQ2020 (scale bar = 200 nm). **(F)** Specific target bands of the cell culture supernatants inoculated with the 5th, 6th 7th 8th, 9th and 10th generation of BRV strain DQ2020s were observed by qPCR. **(G)** Specific target bands of the cell culture supernatants inoculated with the 7th, 8th, 9th, and 10th generation of BRV strain DQ2020s were observed by PCR. **(H)** The colloidal gold immunochromatographic test strip showed no specific positive bands in the cell culture supernatant. **(I)** The colloidal gold immunochromatographic test strip showed specific positive bands of the culture supernatant of cells inoculated with the 10th generation BRV strain DQ2020.

As compared to the control cells, BRV strain DQ2020 induced a remarkable cytopathic effect in MA104 cells at the beginning of passage 8 (P8) (Figures 1A,B). BRV strain DQ2020 carried the VP6 gene as confirmed by the IFA with a monoclonal antibody (Figures 1C,D). BRV-like particles (diameters, ~80 nm) in suspensions of MA104 cells infected with BRV strain DQ2020 (P8) were identified by TEM (Figure 1E). BRV strain DQ2020 carried the VP6 gene as confirmed by qPCR and RT-PCR (Figures 1F,G). A colloidal gold immunochromatographic test strip was used to confirm the presence of BRV (Figures 1H,I).

### Genome sequence of BRV strain DQ2020

The complete genomic sequence of BRV strain DQ2020 (P10) was successfully obtained, and 11 segments (VP1–VP4, VP6, VP7, and NSP1–NSP5) were uploaded to the GenBank database^9^ under the accession numbers PP408165-PP408176. The constellation of this strain was G6P[1]-I2R2C2-M2-A11N2-T6-E2-H3. Based on the nucleotide identity of the coding region sequences, the four gene segments (VP6, VP7, NSP3, and NSP5) were closely related to the cognate genes of BRV strains, with nucleotide sequence identities of 98.60, 96.8, 98.70, and 98.80%, respectively. Six gene segments (VP1, VP2, VP3, VP4, NSP2, and NSP4) were closely related to the cognate genes of human RVA strains, with nucleotide sequence identities of 93.40, 93.50, 96.80, 91.80, 97.20, and 94.40%, respectively. One gene segment (NSP1) was closely related to the cognate genes of a lamb rotavirus strain, with a nucleotide sequence identity of 89.71% (Table 1).

**Table 1:** BRV strain DQ2020 genotyping and strains with the highest nucleotide identity from the GenBank database.

Gene	Closest strain	Nucleotide sequence identify (%)	Amino acid sequence identify (%)	Genotype	Host origin	Nucleotide sequence accession number
VP1	RVA/Human-tc/JPN/AU109/1994/G8P	93.40%	73.64	R2	Human	LC065020
VP2	RVA/Human-wt/HUN/BP1062/2004/G8P	93.50%	77.48	C2	Human	FN665689
VP3	RVA/Human-wt/THA/DB2015-066/2015/G10P	96.70%	88.96	M2	Human	LC367316
VP4	RVA/Human-tc/NGA/HMG035/1999/G8P	91.80%	97.96	P[1]	Human	LC119096
VP6	Sun9	98.60%	92.65	I2	Bovine	AB374146
VP7	RVA/Yak-tc/CHN/QH-1/2015/G6P[1]	96.80%	99.32	G6	Bovine	MK638899
NSP1	CC0812-1/2008	89.71%	87.04	A11	Lamb	HQ834202
NSP2	NT0578	97.20%	88.51	N2	Human	LC060821
NSP3	RVA/Yak-tc/CHN/HY-1/2018/G6P[11]	98.70%	96.92	T6	Bovine	MK250432
NSP4	RVA/Human-wt/VNM/NT0082/2007/G10P[14]	94.40%	96.54	E2	Human	LC095963
NSP5	RVA/Yak-tc/CHN/QH-1/2015/G6P[1]	98.80%	92.57	H3	Bovine	MK638880

### Phylogenetic analysis of genes encoding structural proteins

**Figure 2:**
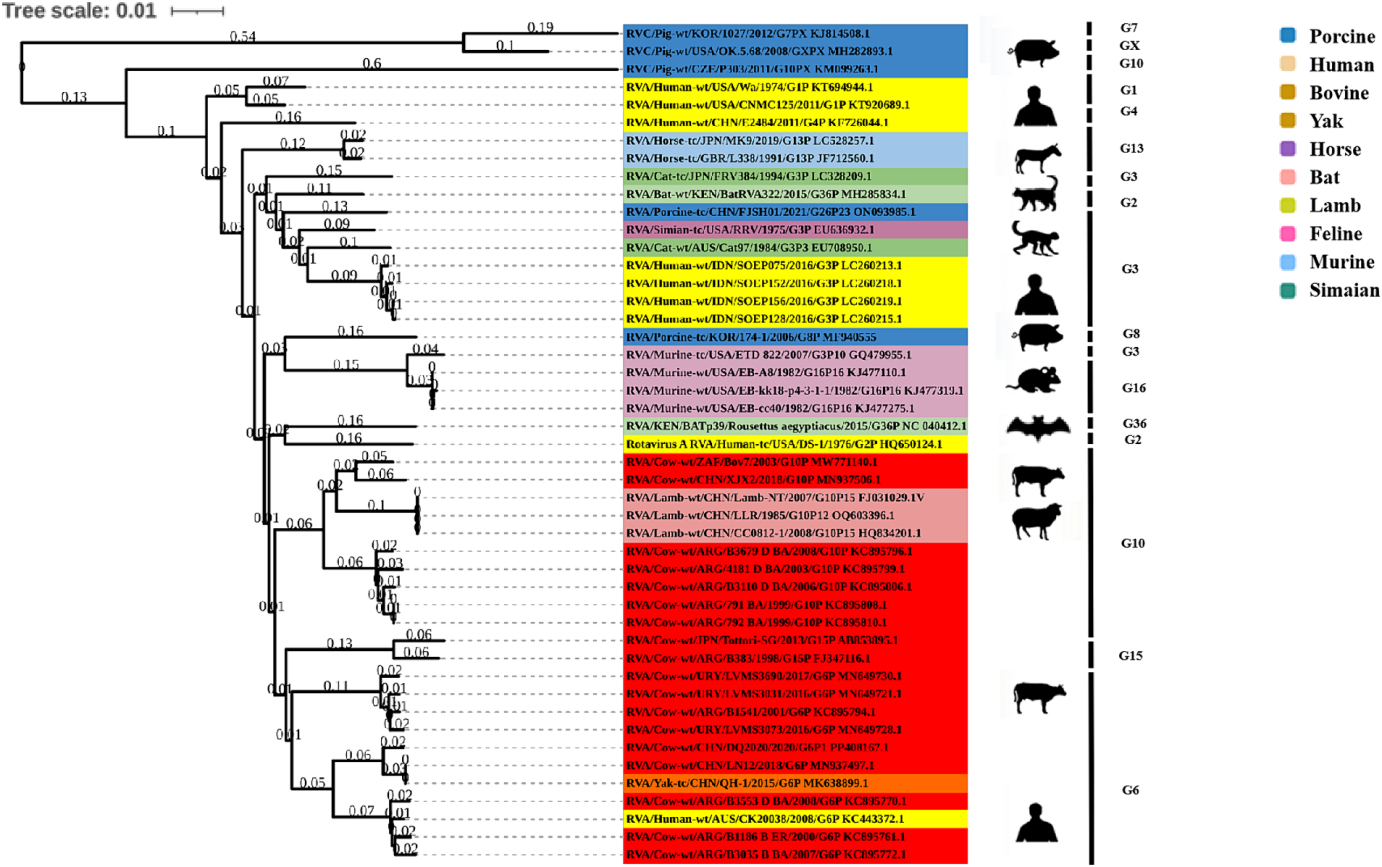
Phylogenetic analysis based on the VP7 gene sequence of BRV.

**Figure 3:**
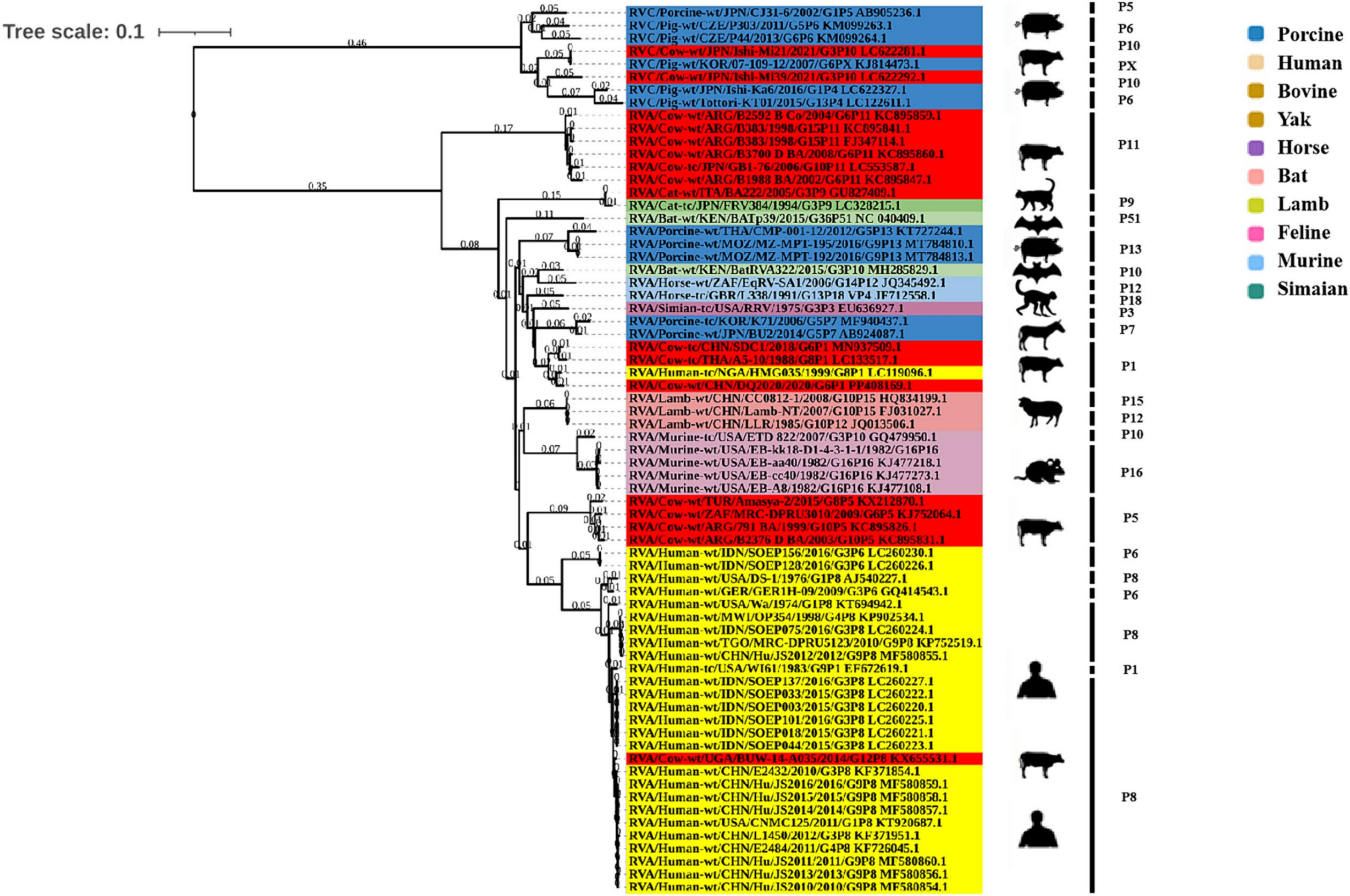
Phylogenetic analysis based on the VP4 gene sequence of BRV.

**Figure 4:**
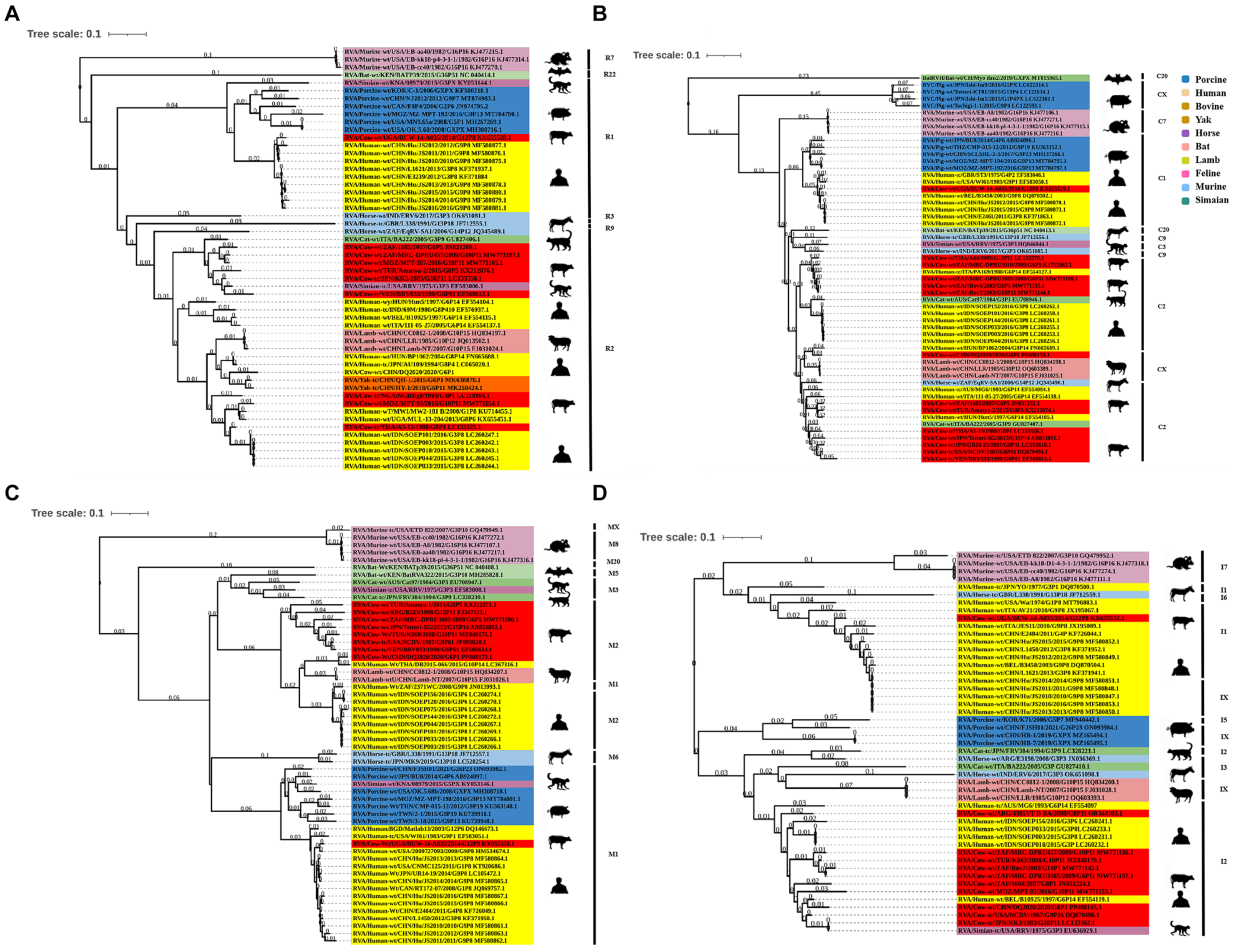
Phylogenetic analysis of other BRV genes coding for structural proteins. **(A–C)** Phylogenetic analysis of the BRV VP1-VP3 genes. **(D)** Phylogenetic analysis of the BRV VP6 gene.

Phylogenetic analysis of the structural genes of strain G6P[1] showed that gene segments VP7 and VP4 were very closely related to a variety of BRV strains with the G6 and P[1] genotypes (Figures 2, 3). The VP7 gene of BRV DQ2020 strain is closely related to the genetic evolution of bovine rotavirus RVA/Yak tc/CHN/QH-1/2015/G6P1 and RVA/Cow tc/CHN/LN12/2018/G6P1 (Figure 2). The VP4 gene of BRV strain DQ2020 was most closely related to the human rotavirus strain RVA/human tc/NGA/HMG035/1999/G8P1 isolated in Nigeria (Figure 3). The VP1 gene of BRV strain DQ2020 clustered with Japanese human strain RVA/Human-tc/JPN/AU109/1994/G8P4 and Hungarian human strain RVA/Human-wt/HUN/BP1062/2004/G8P14 within the R2 genotype. It is interesting to note that BRV DQ2020 was also closed to RVA/Yak-tc/CHN/HY-1/2018/G6P11 and RVA/Yak-tc/CHN/QH-1/2015/G6P1 (Figure 4A). The VP2 gene of BRV strain DQ2020 was closely related to human RVA/human wt/HUN/BP1062/2004/G8P14, both belonging to the C2 type. In addition, both were closely related to the ovine strains LLR, CC0812-1, and Lamb NT isolated in China (Figure 4B). The VP3 gene of BRV strain DQ2020 was most closely related to the human rotavirus strain RVA/human wt/THA/DB2015-066/2015/G10P14 isolated in Thailand, both belonging to the M2 genotype. Also, BRV strain DQ2020 was closely related to human rotaviruses isolated in India (SOEP128, SOEP156, SOEP144, SOEP075, SOEP044, SOEP101, SOEP033, and SOEP003) (Figure 4C). The VP6 gene of BRV strain DQ2020 was closely related to strain RVA/Cow wt/JPN/Sun-9/2008/G8P11 strain and belongs to the I2 genotype. In addition, BRV strain DQ2020 was closely related to strains RVA/Simian-tc/USA/RRV/1975/G3P3 and RVA/Cow tc/JPN/KK3/1983/G10P11 (Figure 4D). These results indicated the genotype of BRV strain DQ2020 were G6, P[1], I2, R2, C2, and M2.

### Phylogenetic analysis of genes encoding non-structural proteins

**Figure 5:**
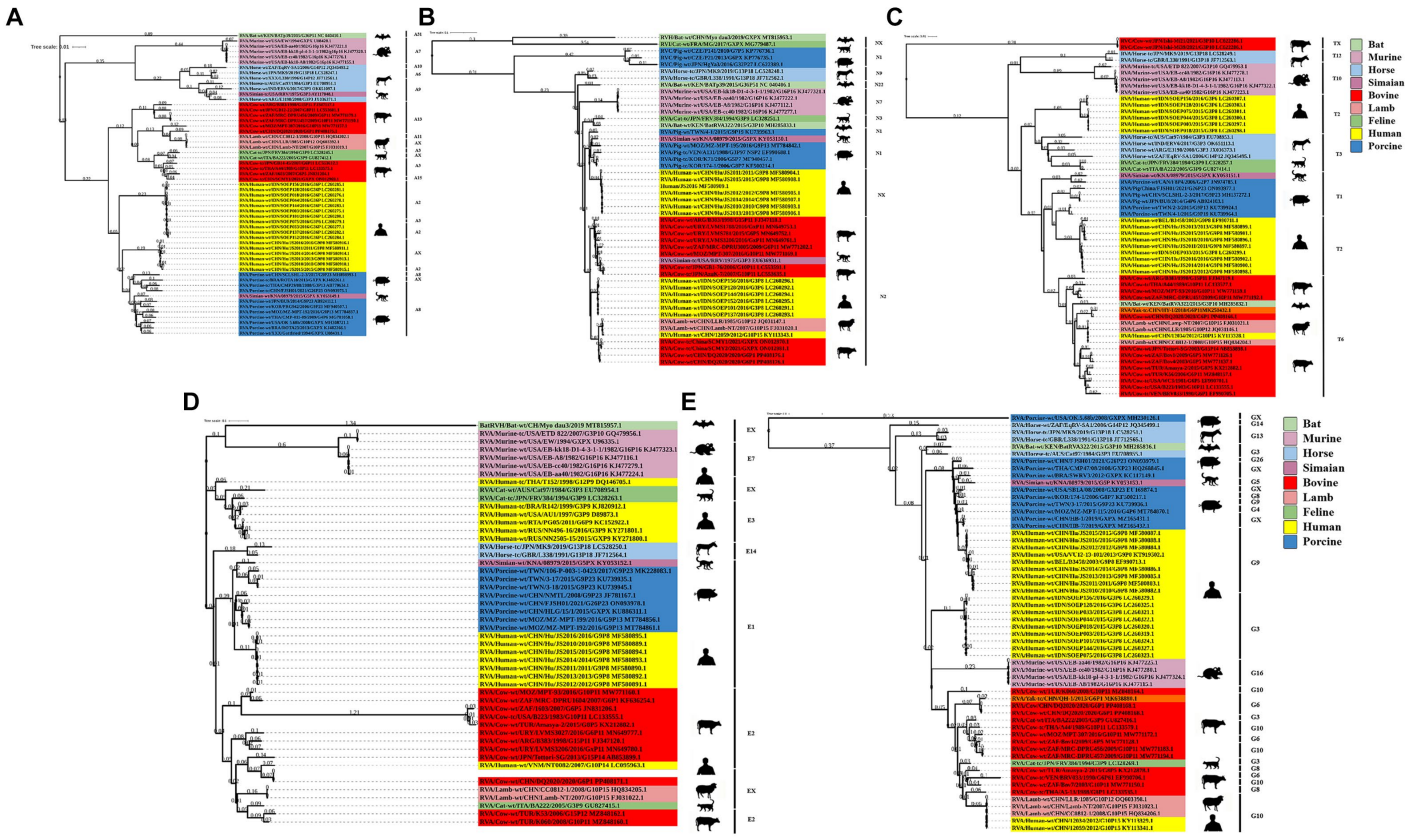
Phylogenetic analysis of BRV genes coding for non-structural proteins. **(A–E)** Phylogenetic analysis of the BRV NSP1-5 genes.

The NSP1 gene of BRV strain DQ2020 was closely related to ovine strains CC0812-1, Lamb NT, and LLR, belonging to the A11 genotype (Figure 5A). The NSP2 gene of BRV strain DQ2020 was most closely related to strain RVA/Cow tc/CHN/SCMY2/2021/GXPX. Interestingly, BRV strain DQ2020 was also closely related to the human strain RVA/human wt/CHN/12059/2012/G10P15, as well as the ovine strains RVA/Lambwt/CHN/LLR/1985/G10P12 and RVA/Lambwt/CHN/LLamb NT/2007/G10P15, both belonging to the N2 genotype (Figure 5B). The NSP3 and NSP5 genes of BRV strain DQ2020 clustered with the bovine strains RVA/Yak tc/CHN/HY-1/2018/G6P11 and RVA/Yak tc/CHN/QH-1/2015/G6P1, belonging to the T6 and H3 genotypes (Figures 5C,E). However, the NSP4 gene was closely related to the human strain RVA/human wt/VNM/NT0082/2007/G10P14, belonging to the E2 genotype. Notably, several nonstructural proteins of BRV strain DQ2020 were similar to the ovine-derived strains CC0812-1, Lamb-NT and LLR. Lastly, the non-structural proteins of BRV strain DQ2020 were closely related to the ovine strains C0812-1, Lamb NT, and LLR (Figure 5D). These results indicated the genotype of BRV strain DQ2020 were A11, N2, T6, E2, and H3.

### Pathogenicity of BRV strain DQ2020 in suckling mice

#### Clinical signs and histopathological analysis

**Figure 6:**
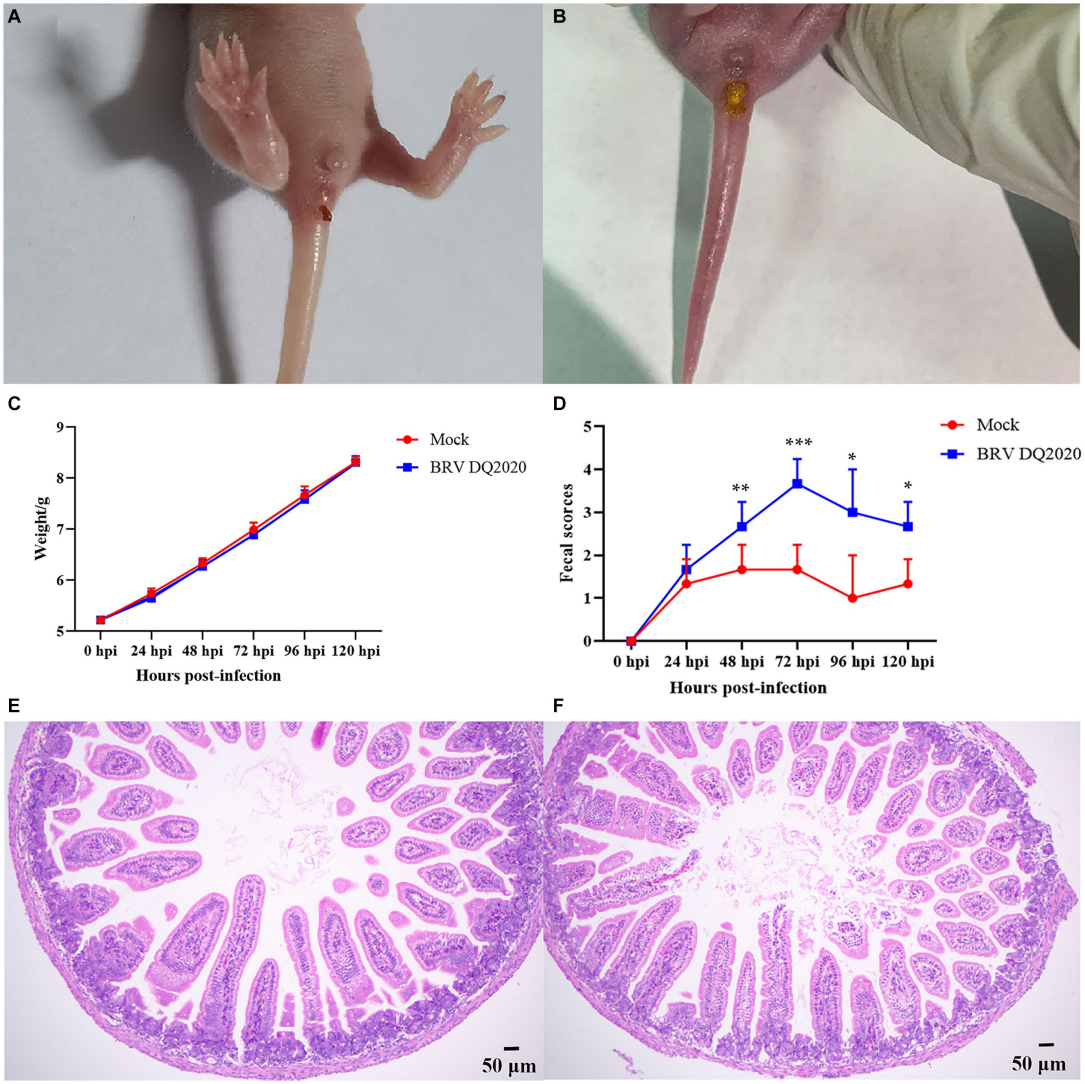
Clinical symptoms and pathological observations of the small intestine tissues of suckling mice. **(A)** The suckling mice inoculated with control medium showed no clinical symptoms. **(B)** Clinical assessment of suckling mice challenged with BRV strain DQ2020. **(C)** The fecal scores of suckling mice in each group. **(D)** The average body weight changes of suckling mice in each group. **(E)** The small intestine tissues of the control mice had normally arranged villi (100×). **(F)** The small intestine tissues of the mice infected with BRV strain DQ2020 exhibited shortening and sloughing of the villi (100×).

In order to clarify the pathogenicity of the BRV DQ2020 strain identified in this study, a pathogenicity test was conducted on BRV DQ2020 infected suckling mice. The results showed that BRV strain DQ2020 induced symptoms of diarrhea, such as soft watery stools with a yellowish color at 48 h post-infection. None of the control mice died or exhibited clinical symptoms of diarrhea (Figures 6A,B).

The pathogenicity results of BRV strain DQ2020 in suckling mice are shown in Figures 6C,D. Of the suckling mice in the experimental group, 3 exhibited mild diarrhea at 24 hpi and all showed severe diarrhea at 72 hpi. Notably, the suckling mice in the experimental group had no significantly body weight changes (*p* > 0.05), as compared to those in the control group (Figures 6C,D). Pathological changes to the small intestine tissues of BRV-infected mice were assessed at 72 h post-infection. The results showed that the small intestine tissues of the control group appeared normal with neatly arranged villi. However, the small intestine tissues of mice infected with BRV strain DQ2020 showed tissue damage, such as villus shedding and atrophy (Figures 6E,F).

#### Measurement of viral load in tissues

**Figure 7:**
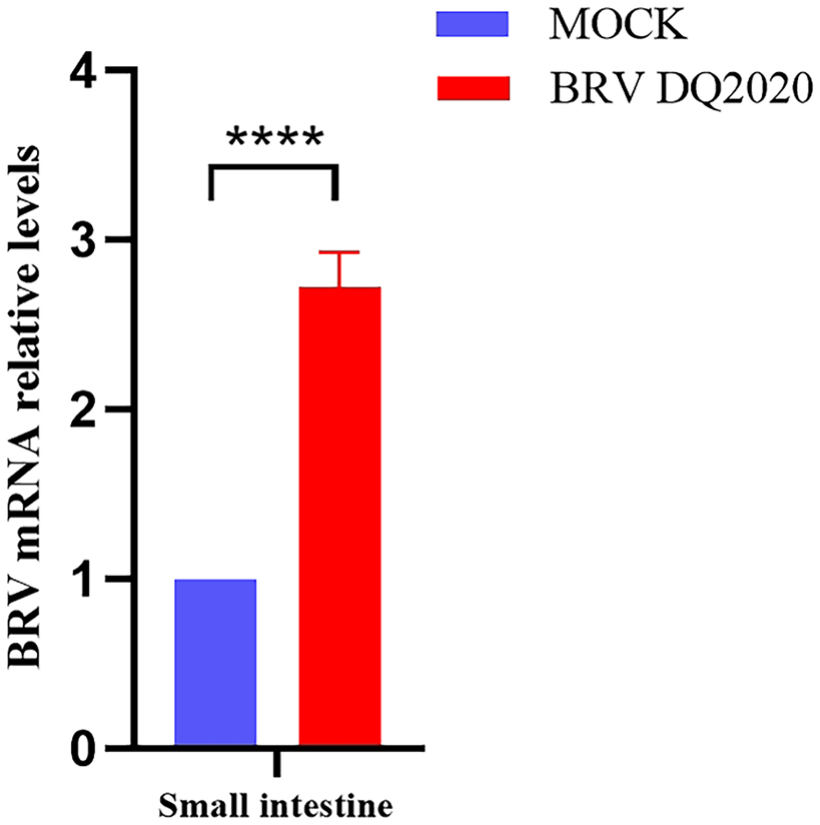
Quantification of viral RNA levels in the small intestine of suckling mice at 72 h inoculated with BRV DQ2020. **** Indicates that the difference was extremely significant. ns indicates no difference between inoculated and control groups.

Viral shedding of the small intestine, rectum, heart, liver, spleen, lung, kidney, and stomach tissues was assessed by RT-qPCR. The results showed that at 72 h post-infection, the BRV mRNA relative levels in the small intestine tissues of suckling mice infected with BRV strain DQ2020 were significantly higher than in control group (Figure 7).

## Discussion

Rotavirus strain A typically infects ruminants and camelids (27–30). BRV is the most common pathogen causing acute diarrhea in calves under 1 month old worldwide, and is also considered a possible pathogen for acute diarrhea in other animals and humans (1). Therefore, BRV not only seriously affects animal health and animal derived food safety, but also poses a potential threat to public health safety. BRV is widely distributed worldwide, with a total infection rate of approximately 20.00 to 70% in different studies. The report shows that from 1993 to 2006, the positive detection rate of BRV in British cattle was 42.00% (2), and in France it was 37.00 to 47.40% (31). The positivity rate of bovine rotavirus in Argentina, South America is 62.50% (32). During 2006 and 2007, the BRV positivity rate in diarrheic calves was at 52.70% in New Zealand (33). A total of 237 calf diarrhea samples were collected from Spanish of which the positive rate for BRV was 13.90% (7). The prevalence of BRV infection in Italian diarrheic calves was 16.8% (34). There have also been reports of BRV testing in some Asian countries, with the prevalence of BRV infection in calf feces in Indian ranging from 11.80 to 26.80% (35). In Australia, the rate of BRV was reported to be 79.90% (36). In China, calf diarrhea outbreaks have been reported in many provinces and regions (37–41). Two G genotypes (G6, G10) and two P genotypes (P[1], P[11]) were identified in the study, with the G6-P[1] genotype BRV being the dominant strain (12). BRV circulates widely among dairy calves in China, and the dominant genotype in circulation is G6P[1] (39). This study successfully isolated the G6-P[1] genotype BRV from bovine diarrhea fecal samples, which has a close genetic evolutionary relationship with other bovine rotavirus isolates in China, and has high similarity in the neutralizing epitope region, suggesting its potential as a vaccine candidate strain.

The complete genome of BRV strain DQ2020 was successfully sequenced. A phylogenetic tree of 11 gene fragments showed that BRV strain DQ2020 was a reassortant of bovine, human, and ovine rotaviruses, but might be genetically mismatched with the human rotavirus strain. Cattle have frequent and close contact with humans, thus increasing the potential risk of cross-species transmission of BRV. Further investigations of the prevalence of BRV in children with diarrhea who have close contact with cattle will help to better understand the public health significance of BRV. Notably, the VP2 gene of BRV strain DQ2020 was closely related to the homologous gene of the ovine strain LLR, which is currently widely used as an attenuated vaccine for children in China (35, 42). The VP2 gene of BRV strain DQ2020 was clustered in the same branch as the vaccine strain. Fragments of the vaccine strain LLR have been reclassified as a human rotavirus. At 2 weeks post-vaccination, the virus was detected in the feces of children and may be transmitted to individuals with weakened immune systems (12, 43–45). Consequently, the rotavirus vaccine strain can be transmitted to animals and cause infection. The findings of this study indicated that the LLR strain might play a role in the evolution of BRV, emphasizing the significance of oral attenuated live vaccines in the evolution of rotaviruses.

Rotavirus VP4 is encoded by genome fragment 4 and is a dimeric, non-glycosylated spike protein located on the surface of virus particles. It is one of the outer shell proteins and the main cross-neutralizing antigen of the virus. It is worth noting that the VP4 protein plays an important role in cell adsorption and penetration. This may also play a crucial role in the adsorption of target receptor cells by different host viruses and cross-species transmission (46). In recent years, a large amount of evidence suggests that BRV strains may be transmitted to humans through cross-species transfer (47). The results of this study indicate that the BRV strain identified may be genetically mismatched with the human rotavirus strain.

VP7 and VP4, respectively, define the G and P genotypes of RVA strains. Thus far, at least 36 G genotypes and 51 P genotypes have been described for strains from humans and animals worldwide.^10^ Previous studies have suggested that the amino acid changes of RVA strains may facilitate viral escape from vaccine-induced immunity because the specific epitopes cannot be recognized (48–50); therefore, the biological significance of this variant needs further investigation. Whether amino acid changes in these strains affect the antigenicity of VP7 and VP4 also needs to be determined. In this study, amino acid sequence comparisons of VP7 and VP4 revealed that there was little difference in amino acid sequence in epitope region between the isolated strain and G6P1 strain prevalent in China (Supplementary Figure S1). The results of this study have a particular reference to the development of a BRV vaccine, as there are still no commercial BoRVA vaccines available in China. The results will contribute to the prevention of dairy calf diarrhea and our understanding of the genetic evolution of BRV in China.

To date, there have been no reports on the pathogenicity of G6P[1] BRV in Heilongjiang Province. Utilizing mice as experimental animals to study the pathogenic mechanism of rotavirus infection (51, 52). Therefore, the viral loads in tissues of mice were quantified to assess the pathogenicity BRV strain DQ2020. The results showed that the inoculated mice produced softer faces than the non-inoculated group and BRV strain DQ2020 was detected in the small intestine tissues of inoculated mice by RT-qPCR. Besides, the inoculated mice developed pathological lesions and atrophy of the small intestine with shortening of the villi and sloughing. In a recent study, 7-day-old BALB/c mice inoculated with rhesus rotavirus by oral gavage developed mild, but consistent, diarrhea at 3–4 days post-infection with notable proliferation of B and T cells (53). Furthermore, BALB/c mice aged 1–2 weeks with “epidemic diarrhea of infant mice” exhibited malaise and reduced activity. Besides, serum and fecal levels of immunoglobulin A were detected and the small intestine tissues exhibited histopathological changes, such as vacuolar degeneration, edema, congestion, and overall integrity destruction (54).

## Conclusion

BRV strain DQ2020 was successfully isolated in Heilongjiang province. The genotype of BRV strain DQ2020 was G6-P[1]-I2-R2-C2-M2-A11-N2-T6-E2-H3, which was a reassortant of human, bovine, and ovine rotaviruses. Notably, the VP4 gene has a close genetic evolutionary relationship with human rotavirus and holds certain public health significance. BRV strain DQ2020 was pathogenic in suckling mice. This study provides a foundation for understanding the genetic variation of BRV and its potential for cross-species transmission and pathogenicity. Additionally, a promising candidate vaccine strain was identified to control BRV outbreaks.

## Data availability statement

The datasets generated in this study can be found in online repositories. The names of the repository/repositories and accession number(s) can be found below: https://www.ncbi.nlm.nih.gov/genbank/, PP408165-PP408176. The datasets generated and/or analyzed during the current study are available in the NCBI Short Read Archive (SRA) with (accession numbers PRJNA1151821).

## Ethics statement

The animal studies were approved by the Animal Experiments Committee of the Heilongjiang Bayi Agricultural University. The studies were conducted in accordance with the local legislation and institutional requirements. Written informed consent was obtained from the owners for the participation of their animals in this study.

## Author contributions

CL: Writing – original draft, Conceptualization, Software. XW: Writing – original draft, Formal analysis, Data curation. QZ: Writing – review & editing, Investigation, Data curation. DS: Writing – review & editing, Funding acquisition, Resources.

## Footnotes

^1^http://soap.genomics.org.cn/

^2^https://github.com/BGI-Qingdao/TGS-GapCloser

^3^http://topaz.gatech.edu/GeneMark/genemarks.cgi

^4^https://blast.ncbi.nlm.nih.gov/Blast.cgi?PAGE=Proteins

^5^https://www.uniprot.org/

^6^http://blast.ncbi.nlm.ni-h.gov/Blast.cgi

^7^http://www.clustal.org/

^8^https://itol.embl.de/

^9^https://www.ncbi.nlm.nih.gov/genbank/

^10^https://rega.kuleuven.be/cev/viralmetagenomics/virus-classification/rcwg
