# Supplementary figures and images for: Isolation and identification of BRV G6P[1] strain in Heilongjiang province, Northeast China
Source: Front Vet Sci. 2024 Sep 20;11:1416465. doi: 10.3389/fvets.2024.1416465 (PMC11449731; doi:10.3389/fvets.2024.1416465)

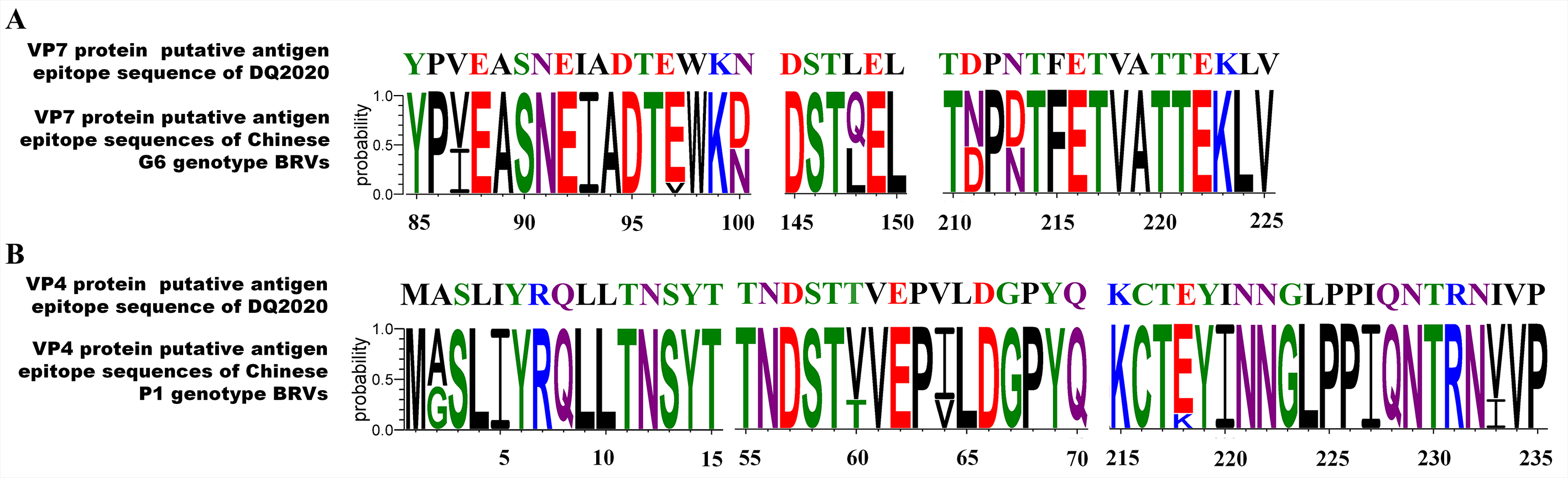

Supplement: Supplementary file 1 [file Image_1.TIF]
