# Supplementary material for: Isolation and identification of BRV G6P[1] strain in Heilongjiang province, Northeast China
Source: Front Vet Sci. 2024 Sep 20;11:1416465. doi: 10.3389/fvets.2024.1416465 (PMC11449731; doi:10.3389/fvets.2024.1416465)
Supplement: Supplementary file 2 [file Table_1.DOCX]

**Table S1.** The sequence of RV reference strains were retrieved from the National Center for Biotechnology Information nucleotide database (GenBank) in this study.

| **Strains name** | **Hosts** | **GenBank accession no.** |
| --- | --- | --- |
| RVA/Porcine-wt/THA/CP29/08/2008/G3P13 | Porcine | AB779634.1 |
| RVA/Simian-wt/USA/RRV/1975/G3P3 | Simian | AY117048.1 |
| RVA/Horese-tc/AUS/Cat97/1984/G3P3 | Horse | EU708951.1 |
| RVA/Cow-wt/ARG/B383/1998/G15P11 | Bovine | FJ347117.1 |
| RVA/Cat-wt/ITA/BA222/2005/G3P9 | Feline | GU827412.1 |
| RVA/Cow-wt/ARG/B2592_B_Co/2004/G6P[11] | Bovine | KC895859.1 |
| RVA/Human-wt/CHN/E3239/2012/G3P[8] | Human | KF371884 |
| RVA/Human-wt/CHN/L1621/2013/G3P8 | Human | KF371941.1 |
| RVA/Human-wt/CHN/L1450/2012/G3P8 | Human | KF371950.1 |
| RVA/Murine-wt/USA/EB-cc40/1932/G16P16 | Murine | KJ477276.1 |
| RVA/Porcine-tc/BRA/ROTA18/2013/GXPX | Porcine | KJ482261.1 |
| RVA/Human-wt/USA/CNMC125/2011/G1P[8] | Human | KT920687.1 |
| RVA/Cow-tc/THA/A5-13/1988/G8P[1] | Human | LC133527.1 |
| RVA/Human-wt/IDN/SOEP033/2016/G3P8 | Human | LC260277.1 |
| RVA/Human-wt/IDN/SOEP156/2016/G3P6 | Human | LC260285.1 |
| RVA/Cat-tc/JPN/FRV384/1994/G3P[9] | Feline | LC328209.1 |
| RVA/Cat-tc/JPN/FRV384/1994/G3P9 | Feline | LC328245.1 |
| RVA/Horese-wt/JPN/MK9/2019/G13P18 | Horse | LC528247.1 |
| RVA/Human-wt/CHN/Hu/JS2011/2011/G9P8 | Human | MF580848.1 |
| RVA/Human-wt/CHN/Hu/JS2011/2011/G9P8 | Human | MF580911.1 |
| RVA/Human-wt/CHN/Hu/JS2013/2013/G9P8 | Human | MF580913.1 |
| RVA/Porcine-w't/MOZ/MZ-MPT-192/2016/G9P13 | Porcine | MT784837.1 |
| RVA/Cow-wt/ZAF/MRC-DPRU456/2009/G6P11 | Bovine | MW771179.1 |
| RVA/Cow-wt/TUR/K063/2008/G10P11 | Bovine | MZ848179.1 |
| RVA/Porcine-wt/CHN/FJSH01/2021/G26P23 | Porcine | ON093984.1 |
| RVA/Porcine-w't/XXX/Gottfried/1994/GXPX | Porcine | U08431.1 |
| EB-aa40/G16P[16] | Murine | KJ477222.1 |
| RVA/Cow-wt/JPN/Tottori-SG/2013/G15P[14] | Bovine | AB853895.1 |
| CJ31-6 | Porcine | AB905214.1 |
| RVA/Pig-wt/JPN/BU2/2014/G5P[7] | Porcine | AB924087.1 |
| RVA/Pig-wt/JPN/BU8/2014/G4P[6] | Porcine | AB924103.1 |
| RVA/Porcine-w't/JPNBU9/2014/G9P23 | Porcine | AB924112.1 |
| RVA/Cow-tc/USA/WC3/1981/G6P[5] | Bovine | ABV66076.1 |
| RVA/Cow-tc/VEN/BRV033/1990/G6P6[1] | Bovine | ABV66077.1 |
| F8P4 | Porcine | AFR33134.1 |
| DS-1 | Human | AJ540227.1 |
| RVA/Cow-wt/TUR/Amasya-2/2015/G8P[5] | Bovine | APZ86782.1 |
| RVA/Simian-tc/USA/RRV/1975/G3P[3] | Simian | AY117048.1 |
| RVA/Cow-tc/USA/B223/1983/G10P[11] | Bovine | BAV35166.1 |
| RVA/Human-wt/AUS/CK20038/2008/G6P[4] | Human | CK20038 |
| SA11-1A | Simian | DQ838634.1 |
| SA11-30/1A (SA11g4Oagentg5delC[30/1A]) | Simian | DQ838644.1 |
| SA11-30/1A | Simian | DQ838649.1 |
| RVA/Cow-tc/USA/NCDV/1967/G9P16 | Bovine | DQ870496.1 |
| RVA/Human-tc/JPN/YO/1977/G3P1 | Human | DQ870500.1 |
| RVA/Human-wt/BEL/B3458/2003/G9P8 | Human | DQ870504.1 |
| RVA/Human-tc/AUS/MG6/1993/G6P14 | Human | EF554097.1 |
| RVA/Human-wt/HUN/Hun5/1997/G6P[14] | Human | EF554104.1 |
| RVA/Human-wt/BEL/B10925/1997/G6P[14] | Human | EF554115.1 |
| RVA/Human-tc/ITA/PA169/1988/G6P[14] | Human | EF554127.1 |
| RVA/Human-wt/ITA/111-05-27/2005/G6P[14] | Human | EF554137.1 |
| RVA/Human-wt/ITA/111-05-27/2005/G6P[14] | Human | EF554138.1 |
| RVA/Sheep-tc/ESP/OVR762/2002/G8P[14] | Lamb | EF554148.1 |
| RVA/Sheep-tc/ESP/OVR762/2002/G8P[14] | Lamb | EF554149.1 |
| RVA/Sheep-tc/ESP/OVR762/2002/G8P[14] | Lamb | EF554150.1 |
| RVA/Sheep-tc/ESP/OVR762/2002/G8P[14] | Lamb | EF554152.1 |
| RVA/Sheep-tc/ESP/OVR762/2002/G8P[14] | Lamb | EF554154.1 |
| RVA/Sheep-tc/ESP/OVR762/2002/G8P[14] | Lamb | EF554155.1 |
| RVA/Sheep-tc/ESP/OVR762/2002/G8P[14] | Lamb | EF554156.1 |
| RVA/Sheep-tc/ESP/OVR762/2002/G8P[14] | Lamb | EF554157.1 |
| RVA/Sheep-tc/ESP/OVR762/2002/G8P[14] | Lamb | EF554158.1 |
| RVA/Cow-tc/VEN/BRV033/1990/G6P6[1] | Bovine | EF560612.1 |
| RVA/Human-tc/IND/69M/1980/G8P4[10] | Human | EF576937.1 |
| RVA/Simian-tc/USA/RRV/1975/G3P[3] | Simian | EF583007.1 |
| RVA/Simian-tc/USA/RRV/1975/G3P[3] | Simian | EF583008.1 |
| RVA/Human-tc/USA/WI61/1983/G9P1A[8] | Human | EF672619.1 |
| RVA/Pig-tc/VEN/A131/1988/G3P9[7] | porcine | EF990688.1 |
| RVA/Human-wt/BEL/B3458/2003/G9P[8] | Human | EF990711.1 |
| RVA/Simian-tc/USA/RRV/1975/G3P[3] | Simian | EU636927.1 |
| RVA/Simian-tc/USA/RRV/1975/G3P[3] | Simian | EU636931.1 |
| RVA/Simian-tc/USA/RRV/1975/G3P[3] | Simian | EU636932.1 |
| Cat97 | Feline | EU708945.1 |
| Cat97 | Feline | EU708946.1 |
| Cat97 | Feline | EU708947.1 |
| Cat97 | Feline | EU708949.1 |
| Cat97 | Feline | EU708950.1 |
| Cat97 | Feline | EU708951.1 |
| Cat97 | Feline | EU708952.1 |
| Cat97 | Feline | EU708953.1 |
| Cat97 | Feline | EU708954.1 |
| Cat97 | Feline | EU708955.1 |
| Lamb-NT | Lamb | FJ031019.1 |
| Lamb-NT | Lamb | FJ031020.1 |
| Lamb-NT | Lamb | FJ031021.1 |
| Lamb-NT | Lamb | FJ031022.1 |
| Lamb-NT | Lamb | FJ031023.1 |
| Lamb-NT | Lamb | FJ031024.1 |
| Lamb-NT | Lamb | FJ031025.1 |
| Lamb-NT | Lamb | FJ031026.1 |
| Lamb-NT | Lamb | FJ031028.1 |
| RVA/Cow-wt/ARG/B383/1998/G15P[11] | Bovine | FJ347114.1 |
| RVA/Cow-wt/ARG/B383/1998/G15P[11] | Bovine | FJ347116.1 |
| RVA/Cow-wt/ARG/B383/1998/G15P[11] | Bovine | FJ347118.1 |
| RVA/Cow-wt/ARG/B383/1998/G15P[11] | Bovine | FJ347119.1 |
| RVA/Human-wt/HUN/BP1062/2004/G8P[14] | Human | FN665688.1 |
| RVA/Human-wt/HUN/BP1062/2004/G8P[14] | Bovine | FN665689.1 |
| GER1H-09 | Human | GQ414543.1 |
| RVA/Murine-tc/USA/ETD_822/2007/G3P10 | Murine | GQ479952.1 |
| ETD_822 | Murine | GQ479953.1 |
| ETD_822 | Murine | GQ479955.1 |
| RVA/Cat-wt/ITA/BA222/2005/G3P[9] | Feline | GU827406.1 |
| RVA/Cat-wt/ITA/BA222/2005/G3P[9] | Feline | GU827407.1 |
| RVA/Cat-wt/ITA/BA222/2005/G3P[9] | Feline | GU827408.1 |
| RVA/Cat-wt/ITA/BA222/2005/G3P[9] | Feline | GU827410.1 |
| RVA/Cat-wt/ITA/BA222/2005/G3P[9] | Feline | GU827412.1 |
| RVA/Cat-wt/ITA/BA222/2005/G3P[9] | Feline | GU827413.1 |
| RVA/Cat-wt/ITA/BA222/2005/G3P[9] | Feline | GU827414.1 |
| RVA/Cat-wt/ITA/BA222/2005/G3P[9] | Feline | GU827415.1 |
| RVA/Cat-wt/ITA/BA222/2005/G3P[9] | Feline | GU827416.1 |
| GO34 | Lamb | GU937877.1 |
| GO34 | Lamb | GU937878.1 |
| GO34 | Lamb | GU937879.1 |
| GO34 | Lamb | GU937881.1 |
| GO34 | Lamb | GU937883.1 |
| GO34 | Lamb | GU937884.1 |
| GO34 | Lamb | GU937885.1 |
| GO34 | Lamb | GU937886.1 |
| GO34 | Lamb | GU937887.1 |
| DS-1 | Human | HQ650124.1 |
| CC0812-1/2008 | Lamb | HQ834200.1 |
| CC0812-1/2008 | Lamb | HQ834202.1 |
| CC0812-1/2008 | Lamb | HQ834203.1 |
| CC0812-1/2008 | Lamb | HQ834204.1 |
| CC0812-1/2008 | Lamb | HQ834205.1 |
| CC0812-1/2008 | Lamb | HQ834206.1 |
| CC0812-1/2008 | Lamb | HQ834207.1 |
| RVA/Horse-tc/GBR/L338/1991/G13P[18] | Horse | JF712555.1 |
| RVA/Horse-tc/GBR/L338/1991/G13P[18] | Horse | JF712556.1 |
| RVA/Horse-tc/GBR/L338/1991/G13P[18] | Horse | JF712557.1 |
| RVA/Horse-tc/GBR/L338/1991/G13P[18] | Horse | JF712558.1 |
| RVA/Horse-tc/GBR/L338/1991/G13P18 | Horse | JF712559.1 |
| RVA/Horse-tc/GBR/L338/1991/G13P[18] | Horse | JF712560.1 |
| RVA/Horse-tc/GBR/L338/1991/G13P[18] | Horse | JF712561.1 |
| RVA/Horse-tc/GBR/L338/1991/G13P[18] | Horse | JF712562.1 |
| RVA/Horse-tc/GBR/L338/1991/G13P[18] | Horse | JF712563.1 |
| RVA/Horse-tc/GBR/L338/1991/G13P[18] | Horse | JF712564.1 |
| RVA/Horse-tc/GBR/L338/1991/G13P[18] | Horse | JF712565.1 |
| RVA/Cow-wt/ZAF/1603/2007/G6P[5] | Bovine | JN831209.1 |
| RVA/Cow-wt/ZAF/1603/2007/G6P5 | Bovine | JN831213.1 |
| F8P4 | Porcine | JN974795.2 |
| LLR | Lamb | JQ013502.1 |
| LLR | Lamb | JQ013503.1 |
| LLR | Lamb | JQ013504.1 |
| LLR | Lamb | JQ031145.1 |
| LLR | Lamb | JQ031146.1 |
| LLR | Lamb | JQ031147.1 |
| LLR | Lamb | JQ031148.1 |
| LLR | Lamb | JQ031150.1 |
| LLR | Lamb | JQ031151.1 |
| RVA/Horse-wt/ZAF/EqRV-SA1/2006/G14P[12] | Horse | JQ345490.1 |
| RVA/Horse-wt/ZAF/EqRV-SA1/2006/G14P[12] | Horse | JQ345492.1 |
| RVA/Horse-wt/ZAF/EqRV-SA1/2006/G14P[12] | Horse | JQ345493.2 |
| RVA/Horse-wt/ZAF/EqRV-SA1/2006/G14P[12] | Horse | JQ345495.1 |
| RVA/Horse-wt/ZAF/EqRV-SA1/2006/G14P[12] | Horse | JQ345499.1 |
| RVA/Horse-wt/ARG/E3198/2008/G3P[3] | Horse | JX036368.1 |
| RVA/Horse-wt/ARG/E3198/2008/G3P3 | Horse | JX036369.1 |
| RVA/Horse-wt/ARG/E3198/2008/G3P[3] | Horse | JX036371.1 |
| RVA/Horse-wt/ARG/E3198/2008/G3P[3] | Horse | JX036373.1 |
| RVA/Human-wt/ITA/AV21/2010/G9P8 | Human | JX195067.1 |
| RVA/Human-wt/ITA/JES11/2010/G9P8 | Human | JX195089.1 |
| RVA/Yak-tc/CHN/QH-1/2015/G6P[1] | Bovine | K638899.1 |
| RVA/Cow-wt/ARG/B1186_B_ER/2000/G6P[5] | Bovine | KC895761.1 |
| RVA/Cow-wt/ARG/B3553_D_BA/2008/G6P[5] | Bovine | KC895770.1 |
| RVA/Cow-wt/ARG/B3035_B_BA/2007/G6P[5] | Bovine | KC895772.1 |
| RVA/Cow-wt/ARG/B1541/2001/G6P[11] | Bovine | KC895794.1 |
| RVA/Cow-wt/ARG/B3679_D_BA/2008/G10P[11] | Bovine | KC895796.1 |
| RVA/Cow-wt/ARG/4181_D_BA/2003/G10P[5] | Bovine | KC895799.1 |
| RVA/Cow-wt/ARG/B3110_D_BA/2006/G10P[11] | Bovine | KC895806.1 |
| RVA/Cow-wt/ARG/791_BA/1999/G10P[5] | Bovine | KC895808.1 |
| RVA/Cow-wt/ARG/792_BA/1999/G10P[5] | Bovine | KC895810.1 |
| RVA/Cow-wt/ARG/791_BA/1999/G10P[5] | Bovine | KC895826.1 |
| RVA/Cow-wt/ARG/B2376_D_BA/2003/G10P[5] | Bovine | KC895831.1 |
| RVA/Cow-wt/ARG/B611_BA/1999/G6P[11] | Bovine | KC895841.1 |
| RVA/Cow-wt/ARG/B1988_BA/2002/G6P[11] | Bovine | KC895847.1 |
| RVA/Cow-wt/ARG/B3700_D_BA/2008/G6P[11] | Bovine | KC895860.1 |
| RVA/Human-wt/CHN/E2432/2010/G3P[8] | Human | KF371854.1 |
| RVA/Human-wt/CHN/L1621/2013/G3P[8] | Human | KF371937.1 |
| RVA/Human-wt/CHN/L1450/2012/G3P[8] | Human | KF371951.1 |
| 174-1 | Porcine | KF500214.1 |
| C-1 | Porcine | KF500218.1 |
| RVA/Human-wt/CHN/E2484/2011/G4P[8] | Human | KF726044.1 |
| RVA/Human-wt/CHN/E2484/2011/G4P[8] | Human | KF726045.1 |
| RVA/Human-wt/CHN/E2484/2011/G4P8 | Human | KF726046.1 |
| EB-A8/G16P[16] | Murine | KJ477106.1 |
| RVA/Murine-wt/USA/EB-A8/1982/G16P16 | Murine | KJ477109.1 |
| EB-A8/G16P[16] | Murine | KJ477110.1 |
| RVA/Murine-wt/USA/EB-A8/1982/G16P16 | Murine | KJ477111.1 |
| EB-A8/G16P[16] | Murine | KJ477112.1 |
| EB-A8/G16P[16] | Murine | KJ477113.1 |
| EB-B8/G16P[16] | Murine | KJ477116.1 |
| EB-B8/G16P[16] | Murine | KJ477122.1 |
| EB-B8/G16P[16] | Murine | KJ477123.1 |
| EB-B8/G16P[16] | Murine | KJ477124.1 |
| EB-B8/G16P[16] | Murine | KJ477125.1 |
| EB-B8/G16P[16] | Murine | KJ477126.1 |
| EB-E7/G16P[16] | Murine | KJ477194.1 |
| EB-aa40/G16P[16] | Murine | KJ477215.1 |
| EB-aa40/G16P[16] | Murine | KJ477216.1 |
| RVA/Murine-wt/USA/EB-aa40/1982/G16P16 | Murine | KJ477221.1 |
| EB-aa40/G16P[16] | Murine | KJ477223.1 |
| EB-cc40/G16P[16] | Murine | KJ477270.1 |
| EB-cc40/G16P[16] | Murine | KJ477271.1 |
| RVA/Murine-wt/USA/EB-cc40/1982/G16P16 | Murine | KJ477274.1 |
| EB-cc40/G16P[16] | Murine | KJ477275.1 |
| EB-cc40/G16P[16] | Murine | KJ477277.1 |
| EB-cc40/G16P[16] | Murine | KJ477278.1 |
| EB-kk18-pl-4-3-1-1/G16P[16] | Murine | KJ477314.1 |
| EB-kk18-pl-4-3-1-1/G16P[16] | Murine | KJ477315.1 |
| RVA/Murine-wt/USA/EB-kk18-D1-4-3-1-1/1982/G16P16 | Murine | KJ477318.1 |
| EB-kk18-pl-4-3-1-1/G16P[16] | Murine | KJ477319.1 |
| RVA/Murine-wt/USA/EB-kk18-p4-3-1-1/1982/G16P16 | Murine | KJ477320.1 |
| EB-kk18-pl-4-3-1-1/G16P[16] | Murine | KJ477321.1 |
| EB-kk18-pl-4-3-1-1/G16P[16] | Murine | KJ477322.1 |
| RVA/Porcine-w't/BRA/ROTA23/2013/GXPX | Porcine | KJ482266.1 |
| RVA/Cow-wt/ZAF/MRC-DPRU3005/2009/G6P[5] | Bovine | KJ751923.1 |
| RVA/Cow-wt/ZAF/MRC-DPRU3010/2009/G6P[5] | Bovine | KJ752062.1 |
| RVA/Cow-wt/ZAF/MRC-DPRU3010/2009/G6P[5] | Bovine | KJ752064.1 |
| RVC/Pig-wt/KOR/07-109-12/2007/G6PX | Porcine | KJ814473.1 |
| RVC/Pig-wt/KOR/1027/2012/G7PX | Porcine | KJ814508.1 |
| RVC/Pig-wt/CZE/P303/2011 | Porcine | KM099263.1 |
| RVC/Pig-wt/CZE/P44/2013 | Porcine | KM099264.1 |
| RVC/Pig-wt/CZE/P59/2013 | Porcine | KM099271.1 |
| RVA/Human-wt/TGO/MRCDPRU5123/2010/G9P[8] | Human | KP752519.1 |
| RVC/Pig-wt/CZE/P21/2013 | Porcine | KP776735.1 |
| RVC/Pig-wt/CZE/P141/2010 | Porcine | KP776736.1 |
| RVA/Human-wt/MWI/OP354/1998/G4P[8] | Human | KP902534.1 |
| RVA/Human-wt/USA/Wa/1974/G1P[8] | Human | KT694942.1 |
| RVA/Human-wt/USA/Wa/1974/G1P[8] | Human | KT694944.1 |
| RVA/Pig-wt/THA/CMP-001-12/2012/G5P[13] | Porcine | KT727244.1 |
| RVA/Human-wt/USA/CNMC125/2011/G1P[8] | Human | KT920689.1 |
| RVA/Human-wt/MWI/MW2-181_B/2000/G1P[8] | Human | KU714455.1 |
| RVA/Pig-wt/TWN/4-1/2015/G9P19 | Porcine | KU739963.1 |
| RVA/Cow-wt/TUR/Amasya-2/2015/G8P[5] | Bovine | KX212870.1 |
| RVA/Cow-wt/TUR/Amasya-2/2015/G8P[5] | Bovine | KX212876.1 |
| RVA/Human-wt/UGA/MUL-13-204/2013/G8P[6] | Human | KX655451.1 |
| RVA/Cow-wt/UGA/BUW-14-A035/2014/G12P[8] | Bovine | KX655528.1 |
| RVA/Cow-wt/UGA/BUW-14-A035/2014/G12P8 | Bovine | KX655537.1 |
| RVA/Simian-wt/KNA/08979/2015/G5P[X] | Simian | KY053144.1 |
| RVA/Simian-wt/KNA/08979/2015/G5P[X] | Simian | KY053145.1 |
| RVA/Simian-wt/KNA/08979/2015/G5P[X] | Simian | KY053149.1 |
| RVA/Simian-wt/KNA/08979/2015/G5P[X] | Simian | KY053150.1 |
| RVA/Simian-wt/KNA/08979/2015/G5P[X] | Simian | KY053151.1 |
| RVA/Simian-wt/KNA/08979/2015/G5P[X] | Simian | KY053152.1 |
| RVA/Simian-wt/KNA/08979/2015/G5P[X] | Simian | KY053153.1 |
| RVA/Pig-wt/UGA/BUW-14-A008/2014/G12P8 | Bovine | KY055419.1 |
| 12059 | Human | KY113343.1 |
| HY-1 | Bovine | KY865389.1 |
| RVA/Human-tc/JPN/AU109/1994/G8P[4] | Human | LC065020.1 |
| RVA/Human-tc/NGA/HMG035/1999/G8P[1] | Human | LC119096.1 |
| RVA/Cow-tc/NGA/NGRBg8/1998/G8P[1] | Bovine | LC119104.1 |
| RVA/Cow-tc/NGA/NGRBg8/1998/G8P[1] | Bovine | LC119105.1 |
| RVA/Cow-tc/NGA/NGRBg8/1998/G8P[1] | Bovine | LC119106.1 |
| RVA/Cow-tc/NGA/NGRBg8/1998/G8P[1] | Bovine | LC119108.1 |
| RVA/Cow-tc/NGA/NGRBg8/1998/G8P[1] | Bovine | LC119110.1 |
| RVA/Cow-tc/NGA/NGRBg8/1998/G8P[1] | Bovine | LC119111.1 |
| RVA/Cow-tc/NGA/NGRBg8/1998/G8P[1] | Bovine | LC119112.1 |
| RVA/Cow-tc/NGA/NGRBg8/1998/G8P[1] | Bovine | LC119113.1 |
| RVA/Cow-tc/NGA/NGRBg8/1998/G8P[1] | Bovine | LC119114.1 |
| RVC/Pig-wt/Tochigi-1-1/2015/G9P[4] | Porcine | LC122593.1 |
| RVC/Pig-wt/Tottori-KT01/2015/G13P[4] | Porcine | LC122611.1 |
| RVC/Pig-wt/Tottori-KT01/2015/G13P[4] | Porcine | LC122614.1 |
| RVA/Cow-tc/THA/A5-13/1988/G8P[1] | Bovine | LC133525.1 |
| RVA/Cow-tc/JPN/KK3/1983/G10P[11] | Bovine | LC133558.1 |
| RVA/Cow-tc/JPN/KK3/1983/G10P11 | Bovine | LC133562.1 |
| RVA/Cow-tc/THA/A44/1989/G10P[11] | Bovine | LC133570.1 |
| RVA/Cow-tc/THA/A44/1989/G10P11 | Bovine | LC133575.1 |
| RVA/Cow-tc/THA/A44/1989/G10P[11] | Bovine | LC133577.1 |
| RVA/Human-wt/IDN/SOEP075/2016/G3P[8] | Human | LC260213.1 |
| RVA/Human-wt/IDN/SOEP128/2016/G3P[6] | Human | LC260215.1 |
| RVA/Human-wt/IDN/SOEP152/2016/G3P[8] | Human | LC260218.1 |
| RVA/Human-wt/IDN/SOEP156/2016/G3P[6] | Human | LC260219.1 |
| RVA/Human-wt/IDN/SOEP003/2015/G3P[8] | Human | LC260220.1 |
| RVA/Human-wt/IDN/SOEP018/2015/G3P[8] | Human | LC260221.1 |
| RVA/Human-wt/IDN/SOEP033/2015/G3P[8] | Human | LC260222.1 |
| RVA/Human-wt/IDN/SOEP044/2015/G3P[8] | Human | LC260223.1 |
| RVA/Human-wt/IDN/SOEP075/2016/G3P[8] | Human | LC260224.1 |
| RVA/Human-wt/IDN/SOEP101/2016/G3P[8] | Human | LC260225.1 |
| RVA/Human-wt/IDN/SOEP128/2016/G3P[6] | Human | LC260226.1 |
| RVA/Human-wt/IDN/SOEP137/2016/G3P[8] | Human | LC260227.1 |
| RVA/Human-wt/IDN/SOEP003/2015/G3P8 | Human | LC260231.1 |
| RVA/Human-wt/IDN/SOEP018/2015/G3P8 | Human | LC260232.1 |
| RVA/Human-wt/IDN/SOEP033/2015/G3P8 | Human | LC260233.1 |
| RVA/Human-wt/IDN/SOEP144/2016/G3P8 | Human | LC260239.1 |
| RVA/Human-wt/IDN/SOEP156/2016/G3P6 | Human | LC260241.1 |
| RVA/Human-wt/IDN/SOEP003/2015/G3P[8] | Human | LC260242.1 |
| RVA/Human-wt/IDN/SOEP018/2015/G3P[8] | Human | LC260243.1 |
| RVA/Human-wt/IDN/SOEP033/2015/G3P[8] | Human | LC260244.1 |
| RVA/Human-wt/IDN/SOEP044/2015/G3P[8] | Human | LC260245.1 |
| RVA/Human-wt/IDN/SOEP101/2016/G3P[8] | Human | LC260247.1 |
| RVA/Human-wt/IDN/SOEP144/2016/G3P[8] | Human | LC260250.1 |
| RVA/Human-wt/IDN/SOEP003/2015/G3P[8] | Human | LC260253.1 |
| RVA/Human-wt/IDN/SOEP033/2015/G3P[8] | Human | LC260255.1 |
| RVA/Human-wt/IDN/SOEP044/2015/G3P[9] | Human | LC260256.1 |
| RVA/Human-wt/IDN/SOEP101/2016/G3P[8] | Human | LC260258.1 |
| RVA/Human-wt/IDN/SOEP152/2016/G3P[8] | Human | LC260262.1 |
| RVA/Human-wt/IDN/SOEP003/2016/G3P8 | Human | LC260275.1 |
| RVA/Human-wt/IDN/SOEP018/2016/G3P8 | Human | LC260276.1 |
| RVA/Human-wt/IDN/SOEP044/2016/G3P8 | Human | LC260278.1 |
| RVA/Human-wt/IDN/SOEP075/2016/G3P6 | Human | LC260279.1 |
| RVA/Human-wt/IDN/SOEP101/2016/G3P8 | Human | LC260280.1 |
| RVA/Human-wt/IDN/SOEP128/2016/G3P6 | Human | LC260281.1 |
| RVA/Human-wt/IDN/SOEP137/2016/G3P8 | Human | LC260282.1 |
| RVA/Human-wt/IDN/SOEP144/2016/G3P8 | Human | LC260283.1 |
| RVA/Human-wt/IDN/SOEP152/2016/G3P8 | Human | LC260284.1 |
| RVA/Human-wt/IDN/SOEP101/2016/G3P[8] | Human | LC260291.1 |
| RVA/Human-wt/IDN/SOEP128/2016/G3P[6] | Human | LC260292.1 |
| RVA/Human-wt/IDN/SOEP137/2016/G3P[8] | Human | LC260293.1 |
| RVA/Human-wt/IDN/SOEP144/2016/G3P[8] | Human | LC260294.1 |
| RVA/Human-wt/IDN/SOEP152/2016/G3P[8] | Human | LC260295.1 |
| RVA/Human-wt/IDN/SOEP156/2016/G3P[6] | Human | LC260296.1 |
| RVA/Human-wt/IDN/SOEP003/2015/G3P[8] | Human | LC260297.1 |
| RVA/Human-wt/IDN/SOEP018/2015/G3P[8] | Human | LC260298.1 |
| RVA/Human-wt/IDN/SOEP033/2015/G3P[8] | Human | LC260299.1 |
| RVA/Human-wt/IDN/SOEP044/2015/G3P[8] | Human | LC260300.1 |
| RVA/Human-wt/IDN/SOEP075/2016/G3P[8] | Human | LC260301.1 |
| RVA/Human-wt/IDN/SOEP128/2016/G3P[6] | Human | LC260303.1 |
| RVA/Human-wt/IDN/SOEP156/2016/G3P[6] | Human | LC260307.1 |
| RVA/Cat-tc/JPN/FRV384/1994/G3P[9] | Feline | LC328215.1 |
| RVA/Cat-tc/JPN/FRV348/1994/G3P[3] | Feline | LC328219.1 |
| RVA/Cat-tc/JPN/FRV317/1994/G3P[9] | Feline | LC328220.1 |
| RVA/Cat-tc/JPN/FRV384/1994/G3P9 | Feline | LC328221.1 |
| RVA/Cat-tc/JPN/FRV348/1994/G3P[3] | Feline | LC328225.1 |
| RVA/Cat-tc/JPN/FRV317/1994/G3P[9] | Feline | LC328226.1 |
| RVA/Cat-tc/JPN/FRV348/1994/G3P[3] | Feline | LC328231.1 |
| RVA/Cat-tc/JPN/FRV317/1994/G3P[9] | Feline | LC328232.1 |
| RVA/Cat-tc/JPN/FRV348/1994/G3P[3] | Feline | LC328237.1 |
| RVA/Cat-tc/JPN/FRV317/1994/G3P[9] | Feline | LC328238.1 |
| RVA/Cat-tc/JPN/FRV348/1994/G3P[3] | Feline | LC328243.1 |
| RVA/Cat-tc/JPN/FRV317/1994/G3P[9] | Feline | LC328244.1 |
| RVA/Cat-tc/JPN/FRV348/1994/G3P[3] | Feline | LC328249.1 |
| RVA/Cat-tc/JPN/FRV317/1994/G3P[9] | Feline | LC328250.1 |
| RVA/Cat-tc/JPN/FRV384/1994/G3P[9] | Feline | LC328251.1 |
| RVA/Cat-tc/JPN/FRV348/1994/G3P[3] | Feline | LC328255.1 |
| RVA/Cat-tc/JPN/FRV317/1994/G3P[9] | Feline | LC328256.1 |
| RVA/Cat-tc/JPN/FRV384/1994/G3P[9] | Feline | LC328257.1 |
| RVA/Cat-tc/JPN/FRV317/1994/G3P[9] | Feline | LC328262.1 |
| RVA/Cat-tc/JPN/FRV348/1994/G3P[3] | Feline | LC328267.1 |
| RVA/Cat-tc/JPN/FRV317/1994/G3P[9] | Feline | LC328268.1 |
| RVA/Horse-tc/JPN/MK9/2019/G13P[18] | Horse | LC528247.1 |
| RVA/Horse-tc/JPN/MK9/2019/G13P[18] | Horse | LC528248.1 |
| RVA/Horse-tc/JPN/MK9/2019/G13P[18] | Horse | LC528249.1 |
| RVA/Horse-tc/JPN/MK9/2019/G13P[18] | Horse | LC528250.1 |
| RVA/Horse-tc/JPN/MK9/2019/G13P[18] | Horse | LC528251.1 |
| RVA/Horse-tc/JPN/MK9/2019/G13P[18] | Horse | LC528255.1 |
| RVA/Horse-tc/JPN/MK9/2019/G13P[18] | Horse | LC528257.1 |
| RVA/Cow-tc/JPN/GB1-76/2006/G10P[11] | Bovine | LC553591.1 |
| RVA/Cow-tc/JPN/GB12-22/2007/G8P11 | Bovine | LC553601.1 |
| RVA/Cow-tc/JPN/GB14-45/2007/G6P11 | Bovine | LC553612.1 |
| RVA/Cow-tc/JPN/AzuK-7/2007/G10P[11] | Bovine | LC553635.1 |
| RVC/Cow-wt/JPN/Ishi-Mi21/2021/G3P10 | Bovine | LC622281.1 |
| RVC/Cow-wt/JPN/Ishi-Mi21/2021/G3P10 | Bovine | LC622286.1 |
| RVC/Cow-wt/JPN/Ishi-Mi39/2021/G3P10 | Bovine | LC622292.1 |
| RVC/Pig-wt/JPN/Ishi-Im1/2015/G1P4PX | Porcine | LC622301.1 |
| RVC/Pig-wt/JPN/Ishi-Im9/2016/G1PX | Porcine | LC622314.1 |
| RVC/Pig-wt/JPN/Ishi-Ka6/2016/G1P4 | Porcine | LC622327.1 |
| RVC/Pig-wt/JPN/HgYa3/2016/GXPX | Porcine | LC622389.1 |
| RVA/Human-wt/CHN/Hu/JS2010/2010/G9P8 | Human | MF580847.1 |
| RVA/Human-wt/CHN/Hu/JS2012/2012/G9P8 | Human | MF580849.1 |
| RVA/Human-wt/CHN/Hu/JS2013/2013/G9P8 | Human | MF580850.1 |
| RVA/Human-wt/CHN/Hu/JS2014/2014/G9P8 | Human | MF580851.1 |
| RVA/Human-wt/CHN/Hu/JS2015/2015/G9P8 | Human | MF580852.1 |
| RVA/Human-wt/CHN/Hu/JS2016/2016/G9P8 | Human | MF580853.1 |
| Hu/JS2010 | Human | MF580854.1 |
| Hu/JS2013 | Human | MF580856.1 |
| Hu/JS2014 | Human | MF580857.1 |
| Hu/JS2015 | Human | MF580858.1 |
| Hu/JS2016 | Human | MF580859.1 |
| Hu/JS2011 | Human | MF580860.1 |
| Hu/JS2011 | Human | MF580876.1 |
| Hu/JS2012 | Human | MF580877.1 |
| Hu/JS2013 | Human | MF580878.1 |
| Hu/JS2014 | Human | MF580879.1 |
| Hu/JS2015 | Human | MF580880.1 |
| Hu/JS2016 | Human | MF580881.1 |
| Hu/JS2010 | Human | MF580882.1 |
| Hu/JS2010 | Human | MF580896.1 |
| Hu/JS2011 | Human | MF580897.1 |
| Hu/JS2012 | Human | MF580898.1 |
| Hu/JS2013 | Human | MF580899.1 |
| Hu/JS2014 | Human | MF580900.1 |
| Hu/JS2015 | Human | MF580901.1 |
| Hu/JS2016 | Human | MF580902.1 |
| Hu/JS2011 | Human | MF580904.1 |
| Hu/JS2012 | Human | MF580905.1 |
| Hu/JS2013 | Human | MF580906.1 |
| Hu/JS2014 | Human | MF580907.1 |
| Hu/JS2015 | Human | MF580908.1 |
| Hu/JS2016 | Human | MF580909.1 |
| RVA/Human-wt/CHN/Hu/JS2010/2010/G9P8 | Human | MF580910.1 |
| RVA/Human-wt/CHN/Hu/JS2014/2014/G9P8 | Human | MF580914.1 |
| RVA/Human-wt/CHN/Hu/JS2015/2015/G9P8 | Human | MF580915.1 |
| RVA/Porcine-tc/KOR/K71/2006/G5P[7] | Porcine | MF940437.1 |
| RVA/Porcine-tc/KOR/K71/2006/G5P7 | Porcine | MF940442.1 |
| RVA/Porcine-tc/KOR/K71/2006/G5P[7] | Porcine | MF940457.1 |
| RVA/Porcine-tc/KOR/PRG942/2006/G9P23 | Porcine | MF940507.1 |
| RVA/Porcine-tc/KOR/174-1/2006/G8P[7] | Porcine | MF940555 |
| RVI/Cat-wt/FRA/MG/2017/GXP | Feline | MG779487.1 |
| RVA/Porcine-w't/THA/CMP-011-09/2009/G4P6 | Porcine | MG781058.1 |
| RVA/Pig-wt/CHN/SCLSHL-2-3/2017/G9P[23] | Porcine | MH137272.1 |
| MN.9.65 | Porcine | MH267269.1 |
| RVC/Pig-wt/USA/OK.5.68/2008 | Porcine | MH282893.1 |
| BatRVA322/Taphozous mauritianus/KEN/Kwale/2015 | Bat | MH285828.1 |
| BatRVA322/Taphozous mauritianus/KEN/Kwale/2015 | Bat | MH285829.1 |
| BatRVA322/Taphozous mauritianus/KEN/Kwale/2015 | Bat | MH285832.1 |
| BatRVA322/Taphozous mauritianus/KEN/Kwale/2015 | Bat | MH285833.1 |
| BatRVA322/Taphozous mauritianus/KEN/Kwale/2015 | Bat | MH285834.1 |
| BatRVA322/Taphozous mauritianus/KEN/Kwale/2015 | Bat | MH285836.1 |
| OK.5.68 | Porcine | MH308716.1 |
| RVA/Porcine-w't/USA/OK5.68b/2008/GXPX | Porcine | MH308721.1 |
| RVA/Porcine-w't/CHN/SCLSHL-2-3/2017/G9P23 | Porcine | MH898993.1 |
| RVA/Yak-tc/CHN/HY-1/2018/G6P[11] | Bovine | MK250424.1 |
| RVA/Yak-tc/CHN/HY-1/2018/G6P[11] | Bovine | MK250425.1 |
| RVA/Yak-tc/CHN/HY-1/2018/G6P[11] | Bovine | MK250426.1 |
| RVA/Yak-tc/CHN/HY-1/2018/G6P[11] | Bovine | MK250428.1 |
| RVA/Yak-tc/CHN/HY-1/2018/G6P[11] | Bovine | MK250430.1 |
| RVA/Yak-tc/CHN/HY-1/2018/G6P[11] | Bovine | MK250431.1 |
| RVA/Yak-tc/CHN/HY-1/2018/G6P[11] | Bovine | MK250432.1 |
| RVA/Yak-tc/CHN/QH-1/2015/G6P[1] | Bovine | MK638870.1 |
| RVA/Yak-tc/CHN/HY-1/2018/G6P[11] | Bovine | MK638876.1 |
| RVA/Yak-tc/CHN/HY-1/2018/G6P[11] | Bovine | MK638879.1 |
| RVA/Yak-tc/CHN/HY-1/2018/G6P[11] | Bovine | MK638880.1 |
| RVA/Cow-wt/URY/LVMS3031/2016/G6P[11] | Bovine | MN649721.1 |
| RVA/Cow-wt/URY/LVMS3073/2016/G6P[11] | Bovine | MN649728.1 |
| RVA/Cow-wt/URY/LVMS3690/2017/G6P[11] | Bovine | MN649730.1 |
| RVA/Cow-wt/URY/LVMS781/2015/G6P[5] | Bovine | MN649752.1 |
| RVA/Cow-wt/URY/LVMS1788/2016/GxP[11] | Bovine | MN649753.1 |
| RVA/Cow-wt/URY/LVMS3206/2016/GxP[11] | Bovine | MN649761.1 |
| RVA/Cow-wt/CHN/XJX2/2018/G10P[X] | Bovine | MN937506.1 |
| RVA/Cow-tc/CHN/SDC1/2018/G6P[1] | Human | MN937523.1 |
| K-98 | Lamb | MT501452.1 |
| K-98 | Lamb | MT501453.1 |
| K-98 | Lamb | MT501454.1 |
| K-98 | Lamb | MT501456.1 |
| K-98 | Lamb | MT501458.1 |
| K-98 | Lamb | MT501459.1 |
| K-98 | Lamb | MT501460.1 |
| K-98 | Lamb | MT501461.1 |
| K-98 | Lamb | MT501462.1 |
| RVA/Pig-wt/MOZ/MZ-MPT-192/2016/G9P[13] | Porcine | MT784790.1 |
| RVA/Pig-wt/MOZ/MZ-MPT-192/2016/G9P[13] | Porcine | MT784797.1 |
| RVA/Pig-wt/MOZ/MZ-MPT-192/2016/G9P[13] | Porcine | MT784805.1 |
| RVA/Pig-wt/MOZ/MZ-MPT-195/2016/G9P[13] | Porcine | MT784810.1 |
| RVA/Pig-wt/MOZ/MZ-MPT-192/2016/G9P[13] | Porcine | MT784813.1 |
| RVA/Pig-wt/MOZ/MZ-MPT-192/2016/G9P[13] | Porcine | MT784821.1 |
| RVA/Pig-wt/MOZ/MZ-MPT-192/2016/G9P[13] | Porcine | MT784837.1 |
| RVA/Pig-wt/MOZ/MZ-MPT-195/2016/G9P[13] | Porcine | MT784842.1 |
| RVA/Pig-wt/MOZ/MZ-MPT-192/2016/G9P[13] | Porcine | MT784845.1 |
| RVA/Pig-wt/MOZ/MZ-MPT-192/2016/G9P[13] | Porcine | MT784853.1 |
| RVA/Pig-wt/MOZ/MZ-MPT-192/2016/G9P[13] | Porcine | MT784869.1 |
| RVA/Human-wt/USA/Wa/1974/G1P8 | Human | MT796883.1 |
| BatRVH/Bat-wt/CH/Myo_dau2/2019 | Bat | MT815950.1 |
| BatRVH/Bat-wt/CH/Myo_dau2/2019 | Bat | MT815953.1 |
| BatRVH/Bat-wt/CH/Myo_dau2/2019 | Bat | MT815957.1 |
| BatRVH/Bat-wt/CH/Myo_dau3/2019 | Bat | MT815963.1 |
| BatRVH/Bat-wt/CH/Myo_dau2/2019 | Bat | MT815965.1 |
| NJ2012 | Porcine | MT874983.1 |
| NJ2012 | Porcine | MT874984.1 |
| NJ2012 | Porcine | MT874985.1 |
| NJ2012 | Porcine | MT874987.1 |
| NJ2012 | Porcine | MT874988.1 |
| NJ2012 | Porcine | MT874989.1 |
| NJ2012 | Porcine | MT874990.1 |
| NJ2012 | Porcine | MT874992.1 |
| NJ2012 | Porcine | MT874993.1 |
| RVA/Cow-wt/ZAF/Bov4/2003/G6P[5] | Bovine | MW771133.1 |
| RVA/Cow-wt/ZAF/Bov7/2003/G10P[11] | Bovine | MW771140.1 |
| RVA/Cow-wt/ZAF/Bov7/2003/G10P1 | Bovine | MW771142.1 |
| RVA/Cow-wt/ZAF/Bov7/2003/G10P[11] | Bovine | MW771144.1 |
| RVA/Cow-wt/MOZ/MPT-93/2016/G10P11 | Bovine | MW771153.1 |
| RVA/Cow-wt/MOZ/MPT-93/2016/G10P[11] | Bovine | MW771154.1 |
| RVA/Cow-wt/MOZ/MPT-307/2016/G10P11 | Bovine | MW771158.1 |
| RVA/Cow-wt/MOZ/MPT-93/2016/G10P[11] | Bovine | MW771159.1 |
| RVA/Cow-wt/MOZ/MPT-307/2016/G10P[11] | Bovine | MW771165.1 |
| RVA/Cow-wt/MOZ/MPT-307/2016/G10P[11] | Bovine | MW771169.1 |
| RVA/Cow-wt/ZAF/MRC-DPRU457/2009/G10P11 | Bovine | MW771186.1 |
| RVA/Cow-wt/ZAF/MRC-DPRU457/2009/G10P[11] | Bovine | MW771187.1 |
| RVA/Cow-wt/ZAF/MRC-DPRU457/2009/G10P11 | Bovine | MW771190.1 |
| RVA/Cow-wt/ZAF/MRC-DPRU457/2009/G10P[11] | Bovine | MW771192.1 |
| RVA/Cow-wt/ZAF/MRC-DPRU3005/2009/G6P11 | Bovine | MW771197.1 |
| RVA/Cow-wt/ZAF/MRC-DPRU3005/2009/G6P[11] | Bovine | MW771202.1 |
| RVA/Porcine-wt/CHN/HB-1/2019/GXPX | Porcine | MZ165494.1 |
| RVA/Porcine-wt/CHN/HB-7/2019/GXPX | Porcine | MZ165495.1 |
| RVA/Cow-wt/JPN/Sun-9/2008/G8P11 | Bovine | MZ314041.1 |
| RVA/Cow-wt/CHN/LN12/2018/G6P[1] | Bovine | N937497.1 |
| BatRVA/KEN/BATp39/Rousettus aegyptiacus/2015 | Bat | NC_040406.1 |
| BatRVA/KEN/BATp39/Rousettus aegyptiacus/2015 | Bat | NC_040408.1 |
| BatRVA/KEN/BATp39/Rousettus aegyptiacus/2015 | Bat | NC_040409.1 |
| RVA/Bat-wt/KEN/BATP39/2015/G36P51 | Bat | NC_040410.1 |
| BatRVA/KEN/BATp39/Rousettus aegyptiacus/2015 | Bat | NC_040412.1 |
| BatRVA/KEN/BATp39/Rousettus aegyptiacus/2015 | Bat | NC_040413.1 |
| BatRVA/KEN/BATp39/Rousettus aegyptiacus/2015 | Bat | NC_040414.1 |
| RVA/Horse-wt/IND/ERV6/2017/G3P[3] | Horse | OK651081.1 |
| RVA/Horse-wt/IND/ERV6/2017/G3P[3] | Horse | OK651093.1 |
| RVA/Horse-wt/IND/ERV6/2017/G3P[3] | Horse | OK651097.1 |
| RVA/Horse-wt/IND/ERV6/2017/G3P3 | Horse | OK651098.1 |
| RVA/Horse-wt/IND/ERV6/2017/G3P[3] | Horse | OK651113.1 |
| RVA/Cow-wt/CHN/SCMY-1/2021/GXPX | Bovine | ON012969.1 |
| RVA/Cow-tc/China/SCMY1/2021 | Bovine | ON012970.1 |
| RVA/Cow-tc/China/SCMY2/2021 | Bovine | ON012981.1 |
| RVA/Porcine-tc/CHN/FJSH01/2021/G26P23 | Porcine | ON093975.1 |
| RVA/Pig/China/FJSH01/2021/G26P[23] | Porcine | ON093977.1 |
| RVA/Pig/China/FJSH01/2021/G26P[23] | Porcine | ON093985.1 |
| RVA/Lamb-wt/CHN/LLR/1985/G10P12 | Lamb | OQ603392.1 |
| RVA/Lamb-wt/CHN/LLR/1985/G10P12 | Lamb | OQ603393.1 |
| LLR | Lamb | OQ603394.1 |
| LLR | Lamb | OQ603395.1 |
| RVA/Murine-wt/USA/EW/1994/GXPX | Murine | U08428.1 |
| RVA/Cow-wt/ZAF/Bov1/2009/G6P[5] | Bovine | UQQ66538.1 |
| RVA/Cow-wt/ZAF/Bov4/2003/G6P[5] | Bovine | UQQ66539.1 |
| RVA/Cow-wt/TUR/K56/2006/G6P[11] | Bovine | UYH99833.1 |
